# PCSK9 promotes the progression and metastasis of colon cancer cells through regulation of EMT and PI3K/AKT signaling in tumor cells and phenotypic polarization of macrophages

**DOI:** 10.1186/s13046-022-02477-0

**Published:** 2022-10-14

**Authors:** Lu Wang, Shuangshuang Li, Huanhua Luo, Qi Lu, Shuwen Yu

**Affiliations:** 1 Department of Pharmacy, Jinan Central Hospital, Shandong University, Jinan, 250012 China; 2 Department of Pharmacy, Central Hospital Affiliated to, Shandong First Medical University, Jinan, 250012 China; 3 Phase I Drug Clinical Trial Center, Qilu Hospital of Shandong University, Jinan, 250012 China; 4 NMPA Key Laboratory for Clinical Research and Evaluation of Innovative Drug, Shandong University, Jinan, 250012 China

**Keywords:** Colon cancer, PCSK9, PI3K, AKT, EMT, Macrophage polarization

## Abstract

**Background:**

Proprotein convertase subtilisin/kexin type 9 (PCSK9) is the ninth member of the proprotein convertase family that regulates lipoprotein homeostasis and altered PCSK9 expression was reportedly associated with tumor development and progression. This study assessed PCSK9 expression and functions in human colon cancer and then explored the underlying molecular events.

**Methods:**

Colon cancer tissues were utilized for analysis of PCSK9 expression for association with clinicopathological factors from patients by immunohistochemistry assay. Manipulation of PCSK9 expression was assessed in vitro and in vivo for colon cancer cell proliferation, migration, and invasion using cell viability CCK-8, Transwell tumor cell migration and invasion, and wound-healing assays. Next, proteomic analysis, Western blot, qRT-PCR and Flow cytometry were conducted to assess downstream targets and tumor cell-derived PCSK9 action on macrophage polarization.

**Results:**

PCSK9 expression was upregulated in colon cancer tissues versus the normal tissues, and associated with advanced tumor pathological grade. Knockdown of PCSK9 expression reduced colon cancer cell proliferation, migration, and invasion and suppressed tumor metastasis in vivo. PCSK9 directly or indirectly upregulated Snail 1 and in turn to downregulate E-cadherin expression, but upregulate N-cadherin and MMP9 levels and thereafter, to induce colon cancer cell epithelial-mesenchymal transition (EMT) process and activated PI3K/AKT signaling. However, PCSK9 overexpression showed the inverse effects on colon cancer cells. Knockdown of PCSK9 expression inhibited M2 macrophage polarization, but also promoted M1 macrophage polarization by reduction of lactate, protein lactylation and macrophage migration inhibitory factor (MIF) levels.

**Conclusion:**

PCSK9 played an important role in the progression and metastasis of colon cancer by regulation of tumor cell EMT and PI3K/AKT signaling and in the phenotypic polarization of macrophages by mediating MIF and lactate levels. Targeting PCSK9 expression or activity could be used to effectively control colon cancer.

**Supplementary Information:**

The online version contains supplementary material available at 10.1186/s13046-022-02477-0.

## Background

Colorectal cancer remains a significant health burden, accounting for 10% of all diagnosed human cancers and 9.4% of cancer-related deaths worldwide in 2020 [1]. The prognosis of metastatic colon cancer is very poor and the median 5-year survival rate is only 18.5% in the United States and 27.7% in Europe [2, 3]. The development of colon cancer is a complex pathological process and involves multiple stages, regulatory factors, and gene alterations, while older age, obesity, tobacco smoke, alcohol consumption, and bad dietary habits are all risk factors in developing colon cancer [4]. To date, surgical resection, radiotherapy, and chemotherapy are the main therapeutic strategies for colon cancer patients [5], while more recently developed targeting therapy and immunotherapy are successfully used to control colorectal cancer [6, 7]. However, despite these treatments, the overall survival and recurrence of metastatic colon cancer patients are still unsatisfactory. Thus, further investigation of colorectal cancer development and progression could help medical oncologists effectively control tumor metastasis and prolong the survival of patients.

Proprotein convertase subtilisin/kexin type 9 (PCSK9), the ninth member of the proprotein convertase family that activates other proteins, is able to bind to the low-density lipoprotein receptor (LDL-R) to regulate lipoprotein homeostasis [8, 9], although it was originally discovered as neural apoptosis-regulated convertase 1 (NARC-1) [10]. PCSK9-inhibitory drugs, like alirocumab and evolocumab, have been approved for trapping circulating PCSK9 for treatment of hypercholesterolemia [11]. PCSK9 is primarily synthetized and expressed in the liver, but it is also expressed in many other tissues, such as the intestinal tract, kidney, brain, heart and the blood vessels [12]. Recent studies demonstrated the role of the cholesterol synthesis pathway in cancer pathogenesis, and cholesterol can participate in the regulation of cancer-related signaling pathways [13, 14] by providing energy for the rapid tumor cell proliferation [15]; however, cholesterol-oxidized derivatives, also known as oxysterols, exhibit a significant apoptotic effect [16–18]. More recently, PCSK9 has been shown to regulate other biological processes in cells, such as cell cycle distribution, inflammation, apoptosis, and carcinogenesis [19–22]. Several recent studies reported that PCSK9 expression was associated with the development and progression of various human cancers, whereas inhibition of PCSK9 expression could attenuate the progression of breast cancer, glioma, hepatocellular cancer, prostate cancer, and lung adenocarcinoma, but promote apoptosis of prostate cancer, glioma, and hepatocellular cancer cells [23]. A recent study published in Nature showed that PCSK9 knockout in mouse cancer cells substantially suppressed their growth in mice in a manner that depends on cytotoxic T cells [24]. Besides, Knockdown of PCSK9 expression suppressed liver metastasis of melanoma B16F1 cells by reduction of serum cholesterol levels [25].

In this study, we first detected PCSK9 expression in colon cancer versus normal tissues for association with clinicopathological characteristics from patients and then assessed the downregulation of PCSK9 expression (*vs.* PCSK9 overexpression) in the regulation of tumor cell proliferation, migration, invasion, and epithelial-mesenchymal transition (EMT) in vitro and tumor cell metastasis in vivo. Then, we assessed the underlying molecular events. This study could offer novel mechanistic insights into the role of PCSK9 in colon cancer development and progression.

## Materials and methods

### Tissue microarray and immunohistochemistry

In this study, we purchased and utilized the commercially available tissue microarray (TMA) slides of colon cancer versus normal tissues (Cat. #HCol-Ade090PG-01; Shanghai Outdo Biotech Co., Ltd, Shanghai, China) for immunohistochemical detection of PCSK9 expression. The TMA contained 30 pairs of colon cancer and corresponding adjacent normal tissues and patients’ information included clinicopathological and survival data (Supplemental Table S1). The study protocol was approved by Shanghai Qut do Biotech Company Ethics Committee (SHYJS-CP-1501006). All experiments were performed in compliance with the relevant regulations, and all patients provided written informed consent.

For immunohistochemistry, TMA were de-paraffinized in xylene and rehydrated in a series of ethanol solutions and into water [26]. Next, the TMA sections were subjected to the antigen retrieval using the PT Link kit (Cat. #YZB/USA0528-2012; Dako North America, Inc., Carpinteria, CA, USA) according to the kit instructions and subsequently, blocked in serum by using the immunohistochemistry KIT (Cat. #KIT-9709, MXB, Fuzhou, China) at the room temperature for 1 h, and then incubated with a primary anti-PCSK9 antibody (Cat. #ab28770; Abcam, Abcam, Cambridge, UK) at a dilution of 1:1000 overnight at 4 °C. On the next day, the sections were washed with TBS thrice and further incubated with a secondary antibody and peroxidase incubation using this immunohistochemistry KIT at the room temperature for 30 min each. The color reaction was then performed using the 3, 3-diaminobenzidine solution and counterstained with hematoxylin. The TBS only was used to replace the primary antibody as a negative control.

The immunostained tissue sections were reviewed and scored by two investigators independently for staining intensity and percentage of tumor cell staining using at least five × 200 magnification fields of each TMA core or animal tissue according to the Fromowitz standard, as described in a previous study [27, 28]. The staining intensity was assessed as 0 (no staining), 1 (weak staining), 2 (moderate staining), and 3 (strong staining), while the percentage of positive tumor cells was divided into four levels, i.e., 1 (0–25% tumor cells stained), 2 (26–50%), 3 (51–75%), and 4 (76–100%). The staining index was obtained by multiplying these two numbers, and a staining index number ≥ 8 was considered high expression, otherwise it was considered low expression.

### Database and data analysis

We searched The Cancer Genome Atlas (TCGA; https://cancergenome.nih.gov/) for PCSK9 expression data on human colon adenocarcinoma (COAD) and normal tissue samples. We then analyzed level of PCSK9 mRNA expression in primary tumor tissues versus normal tissues.

### Cell lines and culture

Human colon cancer cell lines (HCT116 and HT-29) and a human monocyte cell line (THP-1) were purchased from the Chinese Academy of Sciences (Shanghai, China). HCT116 and HT-29 were cultured in McCoy’s 5A medium (KeyGE, Nanjing, China) supplemented with 10% fetal bovine serum (FBS; Biological Industries, Kibbutz Beit-Haemek, Israel), while THP-1 cells were grown in Roswell Park Memorial Institute medium-1640 (RPMI 1640; Gibco, Carlsbad, CA, USA) supplemented with 10% FBS (Gibco) at 37 °C with 5% CO_2_.

### Transient cell transfection

To downregulate PCSK9 expression, we transiently transfected PCSK9 siRNA (IBSBIO, Shanghai, China) into HCT116 and HT-29 cells. In particular, when cells were grown to 30%–50% confluency, scrambled siRNA (used as the negative control) or PCSK9 siRNA was added into the cell culture using Lipofectamine 2000 (Invitrogen, Carlsbad, CA, USA). To overexpress PCSK9, we transiently transfected the PCSK9 plasmid (GenePharma Technologies, Shanghai, China) into 60%–70% confluency of tumor cells, while the empty vector was used as the negative control using Lipofectamine 2000 according to the manufacturer’s instructions. Tumor cells were harvested 24 h after transfection and used for following functional experiments. The sequences of siRNA and plasmid are listed in Supplemental Table S2 and Supplemental Table S3.

### Lentivirus infection

To generate stable PCSK9 knockdown cell lines, we stably infected lentiviral particles carrying GFP-Puro-PCSK9 small hairpin RNA (shRNA) (HanBio, Shanghai, China). In brief, 1.5 × 10^5^ tumor cells were cultured in 24-well plates in 500 µl medium overnight at 37 °C, and then infected with the lentivirus [10 multiplicities of infection (MOI)] when cells were at 30%–50% cell density using 5 µg/ml polybrene (HanBio). The calculation of MOI was MOI = cell number/lentivirus titer (TU/ml) × 1000. Twenty-four hours later, the cell culture medium was removed and 500 µl of the fresh medium was replaced to continue culturing of cells, and then reviewed by using a fluorescence microscope (Olympus, Tokyo, Japan) to determine the infection rate 48–72 h later. The stable cell lines were screened using puromycin (Solarbio, Beijing, China) for 3–4 weeks and used for subsequent experiments after confirmation with qRT-PCR and Western blot for PCSK9 expression.

### Cell viability CCK-8 assay

Change in cell viability was determined by using the Cell Counting Kit-8 (CCK8) assay. In particular, gene-transfected colon cancer cells (at 24 h after transfection) were seeded into 96-well plates at a density of 3000 cells/well in 100 µl of the complete medium and cultured at 37 °C. At the end of each experiment, 10 µl CCK8 reagent (Beyotime, Shanghai, China) was added into each well and the cells were further cultured for 2 h at 37 °C and then the optical density value (OD_450_) was measured by using a Multiskan microplate reader (Thermo Fisher Scientific, Waltham, MA, USA). The assay was in triplicate and repeated at least three times.

### Trypan blue exclusion assay

At 4 days after transfection, cells were prepared into single cell suspension and then mixed with 0.4% trypan blue reagent at a ratio of 9:1, a final concentration of 0.04% was prepared. Within 3 min at 25 °C, a blood cell counting board was used to count live and dead cells under a light microscope (10 × magnification). The experiment was repeated three times.

### Wound-healing assay

The gene transfected cells were plated into 6-well plates with 2 ml culture medium containing 10% FBS and then grown for 24 h. When the cells reached 95–100% confluency, the cells were scratched vertically using a sterile 200 µl pipette tip. The shed cells were then gently washed off with phosphate-buffered saline (PBS), and placed in 2 ml serum-free medium and allowed to grow for 48 h. Cell images at 0 h and 48 h were taken under a light microscope (Olympus, Tokyo, Japan) at a magnification of × 40 in three random fields for each experimental setting. The assay was in done in duplicate and repeated at least three times.

### Analysis of lactate levels

The levels of lactate in the colon caner cell culture supernatant were measured by Lactate Assay Kit-WST (Cat. #L256, Dojindo, Kumamoto, Japan), in accordance with the manufacturer's Research. The assay was repeated at least three times.

### Cell transwell migration and invasion assay

Transwell assay was performed to evaluate the cell migration and invasion ability using Transwell insert chambers with a filter pore size of 8 µm for 24-well plate (Corning Inc., Corning, NY, USA). For the cell migration assay, 1 × 10^5^ cells (HCT116) or 1.5 × 10^5^ cells (HT-29) were seeded into the upper chamber with 200 µl McCoy’s 5A medium without FBS, while 600 µl of the medium containing 15% FBS was placed to the bottom chamber. Cells were incubated at 37 °C for 48 h and the cells remaining at the top side of the filter were removed using a cotton swab. Meanwhile, cells migrating into the reverse side of the filter were fixed with 4% paraformaldehyde (Biosharp, Hefei, China) at room temperature for 20 min and then stained with 0.1% crystal violet (Beyotime, Shanghai, China) for 20 min. After washing off the excess crystal violet with PBS, cells were placed under a microscope and photographed (Olympus, Tokyo, Japan) at a magnification of × 200 in five randomly selected fields and counted.

For the invasion assay, the filter was precoated with 50 µL of Matrigel (BD Bioscience, San Jose, CA, USA), which was diluted with ice-cold serum-free medium at a ratio of 1:8 and then the Transwell chambers were incubated at 37 °C for 1 h. The remaining experimental procedures were identical to the tumor cell Transwell migration assay.

### Co-culture of colon cancer cells with THP-1 derived macrophages

The Transwell chambers with a pore size of 0.4 µm for a 6-well plate (Corning Inc.) were used for the co-culture system of colon cancer cells/THP-1 derived macrophages. In brief, THP-1 cells were seeded in 6-well plates at a density of 7 × 10^5^ cells/well and subsequently treated with 100 ng/ml phorbol 12‐myristate 13‐acetate (PMA; Sigma Chemicals, St. Louis, MO, USA) for 48 h to allow the THP-1 cells to differentiate into macrophages. Meanwhile, colon cancer cells were transfected with PCSK9 siRNA for 24 h and collected and seeded into the upper chamber of the Transwell at a density of 2 × 10^5^ cells/well and incubated overnight to allow attachment. After that, the THP-1-derived macrophages were added into the bottom chambers and co-cultured at 37 °C for 48 h. Next, THP-1-derived macrophages were collected for assaying by using qRT-PCR, Western blot, and flow cytometry.

### The 4D-label-free quantitative proteomics

The 4D-label-free quantitative proteomics was performed by PTM Biolabs (Hangzhou, China) to quantify changes in the whole proteome of colon cancer cell lines. Briefly, HCT116 cells were grown and transfected with siRNA-NC or siRNA-PCSK9 for 48 h and then total cellular protein was extracted for trypsin digestion to obtain peptides. A series of quality controls were used for quality assurance. After that, the company helped us to perform the high-performance liquid chromatography (HPLC) fractionation, liquid chromatography–mass spectrometry (LC–MS)/MS, and then perform a database search, biological information analysis, and the protein–protein interaction construction. The FDR (false discovery rate) for protein identification and peptide spectral matching (PSM) identification was set at 5%, while *p* ≤ 0.05 and the fold-change cutoff of quantitative protein rations more than 1.5 or less than 1/1.5 were considered statistically significant.

### Protein extraction and Western blot

Total cellular protein was extracted from colon cancer cells or THP-1-derived macrophages using the radioimmunoprecipitation assay buffer (RIPA buffer; Beyotime, Shanghai, China) containing protease inhibitor and phosphatase inhibitor after washing the cells twice with ice-cooled PBS for 15 min. The samples were centrifuged at 12,000 rpm at 4 °C for 15 min to collect the supernatants, and the protein concentration was measured using the bicinchoninic acid (BCA) protein assay kit (Beyotime). After that, the protein samples (20 µg each loading) were separated in 8–12% sodium dodecyl sulfate–polyacrylamide gel electrophoresis (SDS-PAGE) gels and then transferred onto the polyvinylidene fluoride (PVDF) membranes (Millipore, Billerica, MA, USA) in an ice bath.

For Western blot, the membranes were incubated in 5% non-fat milk solution in PBS for 2 h at room temperature and then, with primary antibodies against PCSK9 (Cat. #ab181142; Abcam, Cambridge, MA, USA; at a dilution of 1:2000), MMP9 Cat. #13,667; Cell Signaling Technology, Danvers, MA, USA; 1:1000), E-cadherin (Cat. #3195; Cell Signaling Technology; 1:1000), N-cadherin (Cat. #ab76011; Abcam; 1:10,000), Snail 1 (Cat. #sc-271977; Santa Cruz Biotechnology, Santa Cruz, CA, USA; 1:1000), p-PI3K (Cat. #4228; Cell Signaling Technology; 1:1000), AKT (Cat. #9272; Cell Signaling Technology; 1:1000), p-AKT (Cat. #4060; Cell Signaling Technology; 1:2000), L-Lactyl Lysine (Cat. #PTM-1401RM; PTM BIO, Hangzhou, China; 1:1000), MIF (Cat. #MAB289; R&D Systems, Minneapolis, MN, USA; 1:1000), CD163 (Cat. #sc-20066; Santa Cruz Biotechnology; 1:1000), iNOS (Cat. #18,985–1-AP; Peprotech, Rocky Hill, NJ, USA; 1:2000), β-actin (Cat. #66,009–1-Ig; Proteintech; 1:2000) at 4 °C overnight. The following day, the membranes were subsequently incubated with a horseradish peroxidase (HRP)-conjugated secondary goat anti mouse (Cat. #ZB-2305; ZSGB-BIO, Beijing, China; 1:10,000,) or goat anti rabbit antibody (Cat. #ZB-2301; ZSGB-BIO; 1:10,000) at room temperature for 1 h. After that, the protein bands were detected with an enhanced chemiluminescence (ECL) reagent (Millipore) using the FluorChem M System (ProteinSimple, San Francisco, CA, USA) and quantified using ImageJ software (National Institute of Heath, Bethesda, MD, USA) after being normalized to the β-actin level.

### Quantitative reverse transcriptase-polymerase chain reaction (qRT-PCR)

Total cellular RNA was isolated from cells using a TRIzol reagent (Invitrogen) according to the manufacturer’s instructions, quantified using the SpectraMax QuickDrop (Molecular Devices, Silicon Valley, CA, USA), and then reversely transcribed into cDNA using an Evo M-MLV RT Kit with gDNA Clean for qPCR II (Accurate Biotechnology, Changsha, China) following the manufacturer’s protocol. After that, qPCR was performed using an SYBR® Green Premix Pro Taq HS qPCR Kit (Accurate Biotechnology) in the Roche LightCycler® 480 System (Roche, Indianapolis, IN, USA). The amplification conditions were set as follows: 95 °C for 30 s, 95 °C for 5 s, and finally 60 °C for 30 s for 40 cycles. The mRNA level was then normalized to the level of GAPDH mRNA and the relative expression of each mRNA was calculated using the 2^−ΔΔCt^ method. The primer sequences are listed in Supplemental Table S4.

### Flow cytometry

Cells were collected and washed with ice-cold PBS twice to reach single‐cell suspensions in 100 µl of PBS and then stained with 5 µl of AF647‐conjugated CD86 antibody and its isotype (Cat. #305,416; BioLegend, San Diego, CA, USA) at 4 °C for 30 min in the dark according to the manufacturer’s protocol. Next, the cells were washed twice with PBS and resuspended in 300–500 µl of PBS and analyzed using the BD LSRFortessa Cell Analyzer (BD Biosciences) after filtration.

### Animals and experiments

Male, BALB/c, nude, 4–5-week-old mice were purchased from SiPeiFu (Beijing) Biotechnology Co., Ltd (Beijing, China) and housed in individually ventilated cages (IVC) under specific-pathogen-free (SPF) conditions with a 12 h light/dark cycle at 23 ± 1 °C. All animals had free access to standard SPF mouse chow and water. The animal studies were approved by the Animal Ethics Committee of Central Hospital Affiliated to Shandong First Medical University (JNCH2021-79).

In brief, mice were randomly divided into four groups (HCT116-shNC, HCT116-shPCSK9, HT-29-shNC, and HT-29-shPCSK9) for cell injection. Each group consisted of six mice at least. Approximately 5 × 10^6^ cells of HCT116-shNC and HCT116-shPCSK9 groups were subcutaneously injected into the right axillary region of each mouse. The mice were observed at regular intervals then sacrificed after 6 weeks and tumor xenografts and metastasized tumor nodules resected accordingly for further analysis. Meanwhile, approximately 5 × 10^5^ cells of HT-29-shNC and HT-29-shPCSK9 groups in 100 µl FBS-free medium were injected via the tail vein. The mice were observed at regular intervals and sacrificed after 5 weeks for analysis of visible lung surface metastatic nodules. Hematoxylin and eosin (H&E) staining was performed for histological analysis.

### Statistical analysis

All experiments were repeated at least three times and the data were summarized as mean ± SEM and statistically analyzed using SPSS 19.0 (SPSS, Chicago, IL, USA) and GraphPad Prism 8.0 software (GraphPad Software, La Jolla, CA, USA). The difference between the two groups was determined by using Student’s *t*-test, while the difference among multiple groups was assessed by using the one-way analysis of variance (ANOVA) test. The correlation between PCSK9 expression and clinicopathological characteristics was analyzed by the Chi-squared (χ^2^) test; *p* < 0.05 was considered a statistically significant difference.

## Results

### Upregulation of PCSK9 expression in colon cancer tissues and association of PCSK9 expression with clinicopathological factors from patients

**Fig. 1:**
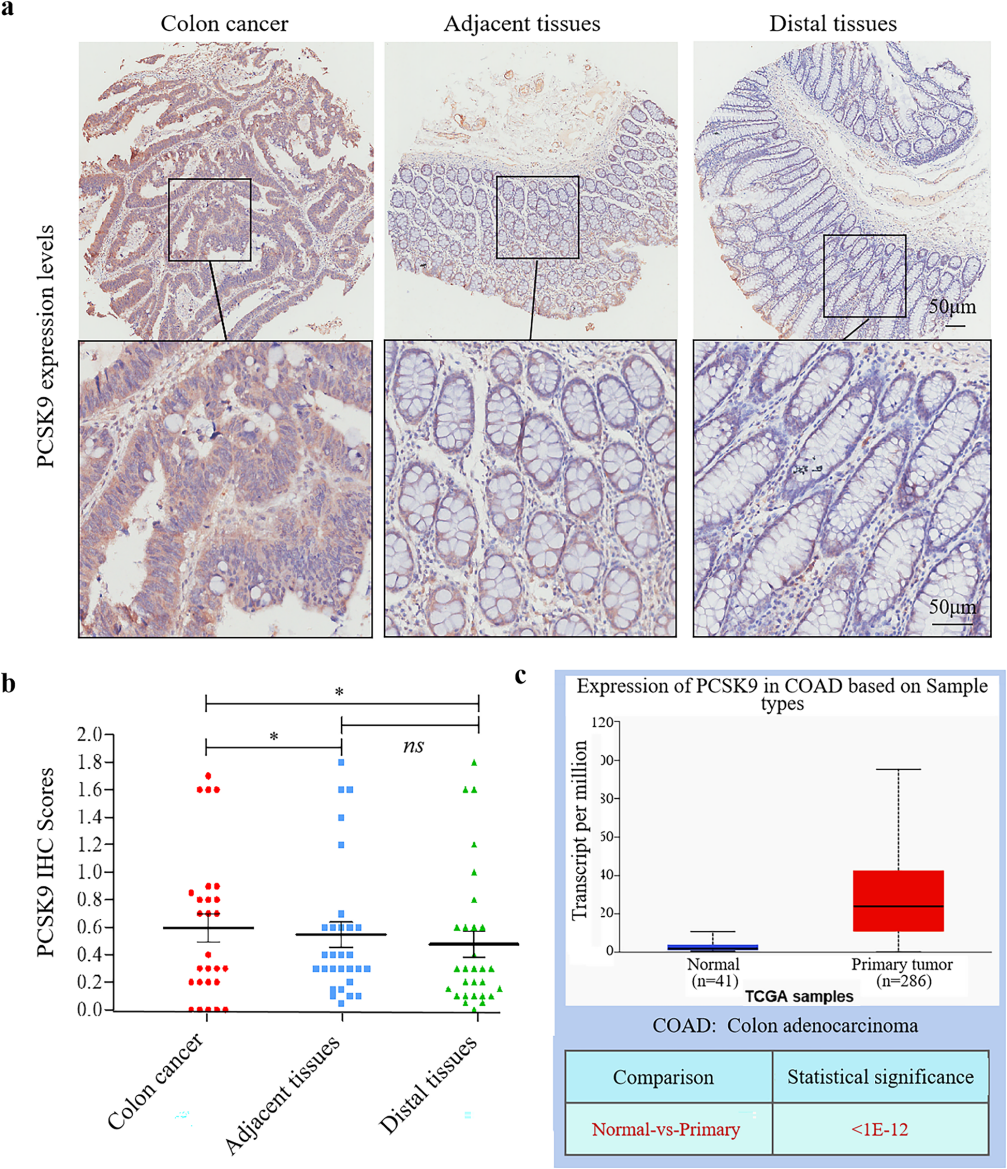
Upregulated PCSK9 expression in colon cancer tissues. **a**, Immunohistochemical analysis of PCSK9 protein in colon cancer tissue microarray. **b**, Quantified data of immunohistochemistry. **c**, PCSK9 expression using TCGA dataset of 41 normal tissues and 286 colon adenocarcinoma tissues. *p < 0.05; *ns*, no significance

In this study, we first detected PCSK9 protein expression in colon cancer tissues and found that PCSK9 protein was mainly expressed in the cytoplasm of tumor cells at a high level compared with that of the corresponding adjacent normal tissues and distal tissues, while the level of PCSK9 expression was similar between the adjacent and distal tissues (Fig. 1a and b and Table 1). After that, we analyzed PCSK9 level using the TCGA data (which included 41 normal tissues and 286 colon adenocarcinoma tissues) and found that PCSK9 expression was significantly higher in the primary tumor samples than that in the normal tissues (Fig. 1c), which is consistent with our conclusion.

**Table 1:** PCSK9 expression in colon cancer tissues and the corresponding adjacent and distal normal tissues

	PCSK9 expression		χ^2^ value	*p* value
	High (%)	Low (%)		
Colon cancer	50	50	5.438	0.020 ^*^
Adjacent tissues	19.2	80.8		
Colon cancer	50	50	4.064	0.044 ^*^
Distal tissues	23.1	76.9		
Adjacent tissues	19.2	80.8	0.115	0.73
Distal tissues	23.1	76.9		

*Note*: ^*^p < 0.05

After that, we associated PCSK9 expression with clinicopathological data from the patients and found that PCSK9 expression was associated with advanced pathological grade of tumor. In grade I/II, there are more colon caner patients with low expression of PCSK9 in tumor cells. Moreover, there was no association of PCSK9 with the age, gender, tumor size, N stage, and TNM stage of the patients (Table 2).

**Table 2:** Association of PCSK9 expression with clinicopathological parameters from colon cancer patients

Variables		PCSK9 expression		n	χ^2^ value	*p* value
		Low	High			
Age (years)					0.054	0.81
	≤ 55	8	8	16		
	> 55	6	5	11		
Sex					1.192	0.27
	Female	9	10	19		
	Male	5	2	7		
Grade					4.338	0.037 ^*^
	I/II	13	7	20		
	III	1	5	6		
N stage					0.074	0.78
	N0	5	4	9		
	N1/N2	9	9	18		
TNM stage					0.074	0.78
	Ι/II	5	4	9		
	III	9	9	18		
Tumor size (cm)					0.326	0.56
	≤ 4	8	6	14		
	> 4	6	7	13		

*Note*: *n* number of cases

^*^ *p* < 0.05

### Reduction of colon cancer cell viability, migration, and invasion after knockdown of PCSK9 expression in vitro

**Fig. 2:**
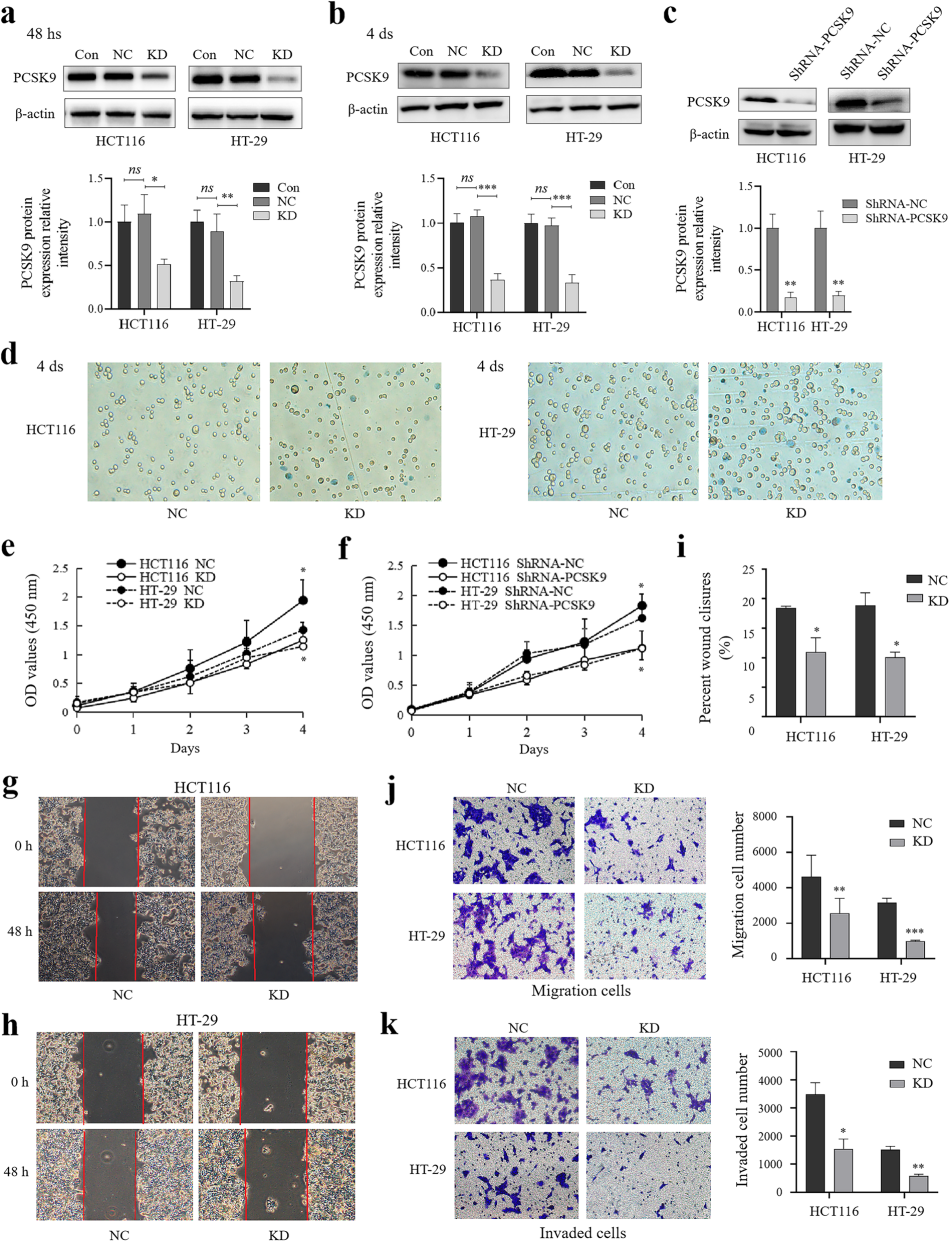
Reduction of colon cancer cell proliferation, migration, and invasion capacities after the reduction of PCSK9 expression in vitro. **a-c**, Western blot. HCT116 and HT-29 cells were grown and transfected with PCSK9 siRNA or PCSK9 shRNA and then subjected to Western blot analysis of PCSK9 expression. The graph is quantified data of Western blots (*n* = 3). **d **and** e**, Trypan blue exclusion assay and CCK-8 assay. Knockdown of PCSK9 expression decreased the viability of HCT116 and HT-29 cells at day 4 after transient transfaction. **f**, CCK-8 assay. Knockdown of PCSK9 expression decreased the proliferation of stable transfected cells at day 4 after seeding. **g**-**i**, Wound-healing assay. HCT116 and HT-29 cells were grown and transiently transfected with PCSK9 or control siRNA and then subjected to the assay. The graph is the quantified data of the assay. The data showed that PCSK9 silence reduced tumor cell wound healing rates of HCT116 and HT-29**.** Magnification, × 40. **j**, Transwell assay. HCT116 and HT-29 cells were grown and transiently transfected with PCSK9 or control siRNA, and then subjected to the Transwell assay. The data revealed that PCSK9 knockdown inhibited migration of HCT116 and HT-29 cells. Magnification, × 200. **k**, Transwell invasion assay. HCT116 and HT-29 cells were grown and transiently transfected with PCSK9 or control siRNA, and then subjected to the Transwell assay. The data showed that PCSK9 knockdown inhibited the invasion of HCT116 and HT-29 cells. Magnification, × 200. Con, control cells without transfection; NC, negative control cells transfected with negative control siRNA; KD, knockdown of PCSK9 cells transfected with PCSK9 siRNA. *ns,* no significance; hs, hours; ds, days. *p < 0.05; **p < 0.01; ***p < 0.001

We then assessed the in vitro effects of PCSK9 knockdown on the regulation of colon cancer cell phenotypes. We transiently transfected PCSK9 siRNA versus the negative control (NC) siRNA into HT-29 and HCT116 cells and stably infected cells with the lentiviral particles carrying PCSK9 shRNA to generate stable PCSK9 knockdown sublines. Western blot data confirmed reduction of PCSK9 protein expression (Fig. 2a-c). Besides, we performed cell viability CCK-8, Trypan blue exclusion assay, Transwell tumor cell migration and invasion, and wound-healing assays. Our data revealed that knockdown of PCSK9 expression reduced the viability of HCT116 and HT-29 cells at day 4 after transient transfaction by using CCK-8 and Trypan blue exclusion assay (all *p* < 0.05) (Fig. 2d and e). Knockdown of PCSK9 expression decreased the proliferation of stable transfected cells at day 4 after seeding (Fig. 2f). The wound-healing assays (Fig. 2g-i) and Transwell data (Fig. 2j and k) revealed that PCSK9 knockdown reduced the capacity of HCT116 and HT-29 cell migration and invasion.

Moreover, for transient transfection, the protein is decreased by 66.1% for HCT116 and 65.9% for HT-29 and by using the viral vector, the protein is decreased by 82.8% for HCT116 and around 80.3% for HT-29. However, such a decrease in PCSK9 protein expression by using the viral vector did not impact cell proliferation in a more clear cut way. Therefore, we speculated that PCSK9 regulation might not directly affect cell survival, but in some way indirectly.

### Promotion of colon cancer cell proliferation, migration, and invasion after PCSK9 overexpression in vitro

**Fig. 3:**
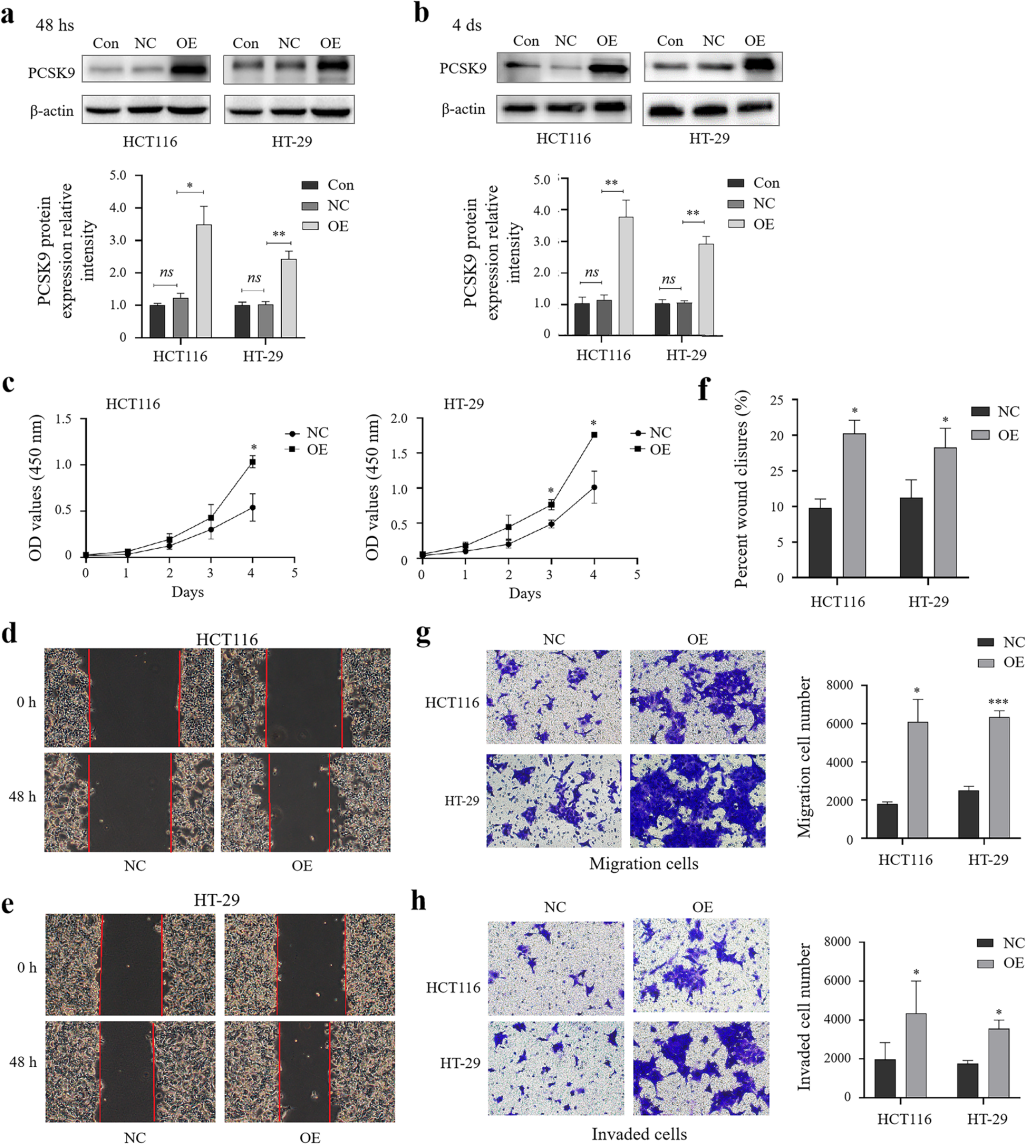
Promotion of colon cancer cell proliferation, migration, and invasion after transfection in vitro. **a-b**, Western blot. HCT116 and HT-29 cells were grown and transiently transfected with plasmids carrying PCSK9 cDNA or vector only, and then subjected to Western blot analysis of PCSK9 expression at 48 hs or day 4 after transfaction. The graph is quantified data of Western blots (*n* = 3). **c**, Cell viability assay. HCT116 and HT-29 cells were grown and transiently transfected with plasmids carrying PCSK9 cDNA or vector only, and then subjected to CCK8 assay. The data showed that PCSK9 overexpression enhanced proliferation of HCT116 and HT-29 cells. **d–f**, Wound-healing assay. HCT116 and HT-29 cells were grown and transiently transfected with plasmids carrying PCSK9 cDNA or vector only, and then subjected to the assay. The data showed that PCSK9 overexpression induced wound-healing rates of HCT116 and HT-29 cells. Magnification, × 40. The graph is quantified data. **g **and** h**, Transwell assay. HCT116 and HT-29 cells were grown and transiently transfected with plasmids carrying PCSK9 cDNA or vector only, and then subjected to Transwell assays. The graphs are quantified data. The data revealed that enforced PCSK9 expression promoted tumor cell migration (**g**) and invasion capacities (**h**) of HCT116 and HT-29 cells. Magnification, × 200. Con, control cells without transfection; NC, negative control cells transfected with plasmids carrying vector; OE, overexpression of PCSK9 cells transfected with plasmids carrying PCSK9 cDNA. *ns,* no significance; hs, hours; ds, days. *p < 0.05; **p < 0.01; ***p < 0.001

To further confirm the oncogenic effects of PCSK9 expression in colon cancer cells, we over-expressed PCSK9 in HCT116 and HT-29 cells transiently. Our Western blot data showed that PCSK9 expression was upregulated in these cells compared to that of cells transfected with a vector only (Fig. 3a and b). We then assessed changes in tumor cell proliferation, migration and invasion and found that PCSK9 overexpression did have the opposite effects on colon cancer cells in vitro (Fig. 3c-h).

### The epithelial-mesenchymal transition (EMT) of colon cancer cells manipulated by PCSK9 expression

**Fig. 4:**
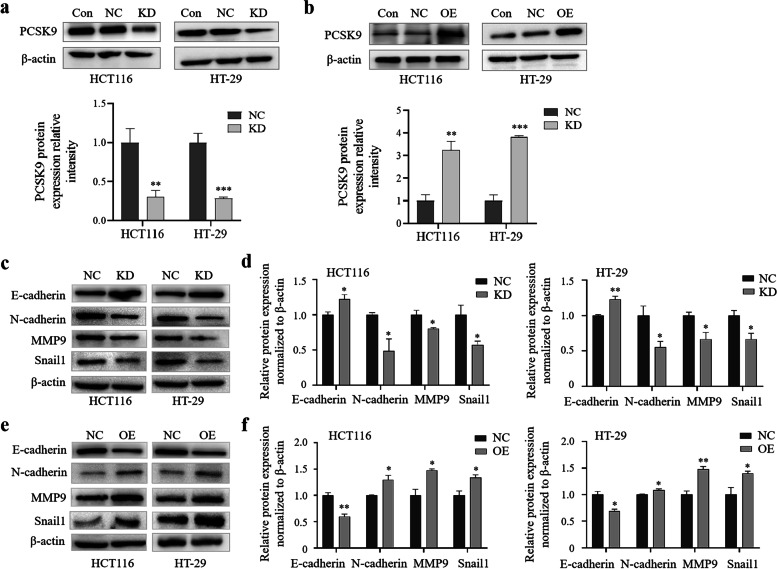
Expression of colon cancer cell EMT-related proteins after manipulation of PCSK9 expression in vitro. **a**,** c **and** d**, Western blot. HCT116 and HT-29 cells were grown and transiently transfected with PCSK9 or control siRNA, and then subjected to Western blot analysis of protein expression at 48 hs after transfaction. The graph is quantified data of Western blots. The data revealed that PCSK9 knockdown increased level of E-cadherin protein, but reduced N-cadherin, MMP9, and Snail 1 proteins. **b**,** e **and** f**, Western blot. HCT116 and HT-29 cells were grown and transiently transfected with plasmids carrying PCSK9 cDNA or vector only, and then subjected to Western blot analysis of protein expression. The graph is quantified data of Western blots. The data showed that PCSK9 overexpression decreased level of E-cadherin protein, but increased level of N-cadherin, MMP9, and Snail 1 proteins. NC, negative control; KD, knockdown of PCSK9 cells transfected with PCSK9 siRNA; OE, overexpression of PCSK9 cells transfected with plasmids carrying PCSK9 cDNA. *p < 0.05; **p < 0.01; ***p < 0.001

Firstly, the representative western blots of proteins showed that PCSK9 expression was significantly down-regulated or up-regulated in PCSK9-siRNA transfected cells or PCSK9 over-expressed cells, compared to that of control cells ( Fig. 4a and b). To better understand the effects of PCSK9 knockdown in colon cancer cells, we analyzed the levels of the EMT process-related proteins. As we know, the EMT is a process during which epithelial cells acquire the phenotype of the mesenchymal stem cells under specific physiological and pathological conditions and importantly, EMT mediates colorectal cancer progression and metastasis [29–32]. We therefore detected the changes in such protein expressions in colon cancer cells (i.e., knockdown of PCSK9 expression significantly upregulated level of E-cadherin protein), but downregulated expressions of N-cadherin, MMP9, and Snail 1 proteins in HCT116 and HT-29 cells (Fig. 4c and d). In contrast, PCSK9 overexpression suppressed the expression of E-cadherin protein, but upregulated expressions of N-cadherin, MMP9, and Snail 1 proteins in HCT116 and HT-29 cells (Fig. 4e and f). These data suggest that PCSK9 expression altered colon cancer cell EMT, thereby changing tumor cell migration and invasion capacities.

### PCSK9 regulation of colon cancer progression through the PI3K/AKT signaling

**Fig. 5:**
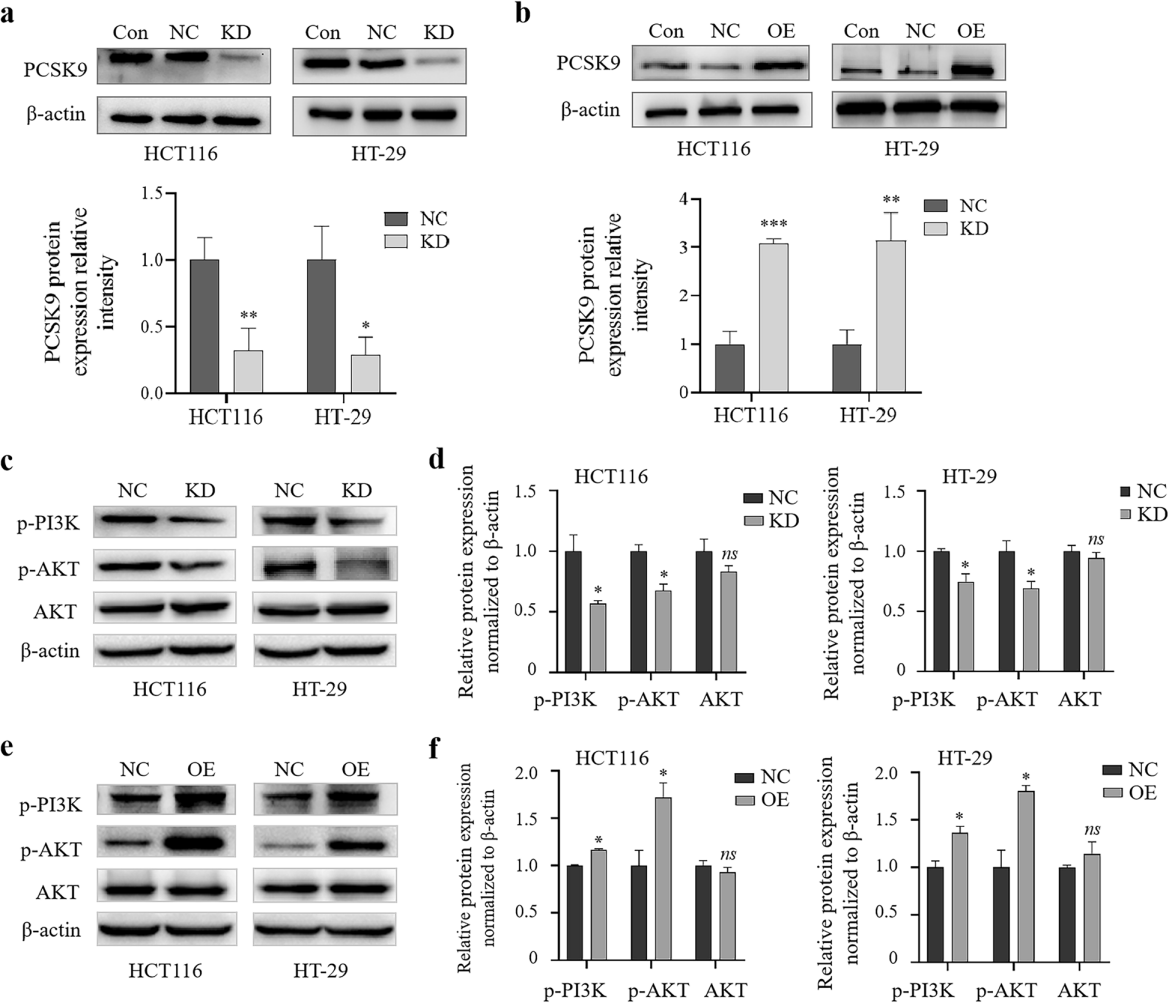
PCSK9 activation of the PI3K/AKT signaling in colon cancer cells in vitro. **a–f**, Western blot. HCT116 and HT-29 cells were grown and transiently transfected with PCSK9 or control siRNA or with plasmids carrying PCSK9 cDNA or vector only, and then subjected to Western blot analysis of p-PI3K, p-AKT, and AKT expression (**c **and** d**, PCSK9 knockdown; **e **and** f**, PCSK9 overexpression). NC, negative control; KD, knockdown of PCSK9 cells transfected with PCSK9 siRNA; OE, overexpression of PCSK9 cells transfected with plasmids carrying PCSK9 cDNA. *p < 0.05, **p < 0.01; ***p < 0.001; *ns,* no significance

As we know, the phosphatidylinositol 3-kinase/Protein Kinase-B (PI3K/AKT) signaling pathway is one of the most commonly activated pathways in human cancers by promoting tumor cell survival, proliferation, metabolism, invasion, and angiogenesis [33, 34]. Thus, in this study, we assessed the changes in expression of this signaling proteins. The representative western blots of proteins showed that PCSK9 expression was significantly down- or up-regulated in PCSK9-siRNA or PCSK9 over-expressed cells, compared to that of control cells (Fig. 5a and b). Then, downregulation of PCSK9 indeed decreased levels of p-PI3K and p-AKT proteins, but did not significantly affect the total AKT levels in HCT116 and HT-29 cells (Fig. 5c and d). However, PCSK9 overexpression induced expression of p-PI3K and p-AKT proteins, but also did not significantly affect the total AKT levels in HCT116 and HT-29 cells (Fig. 5e and f).

### Proteomic analysis of the regulatory effects of PCSK9 on immunity and metabolism of colon cancer cells and macrophage phenotypic polarization

**Fig. 6:**
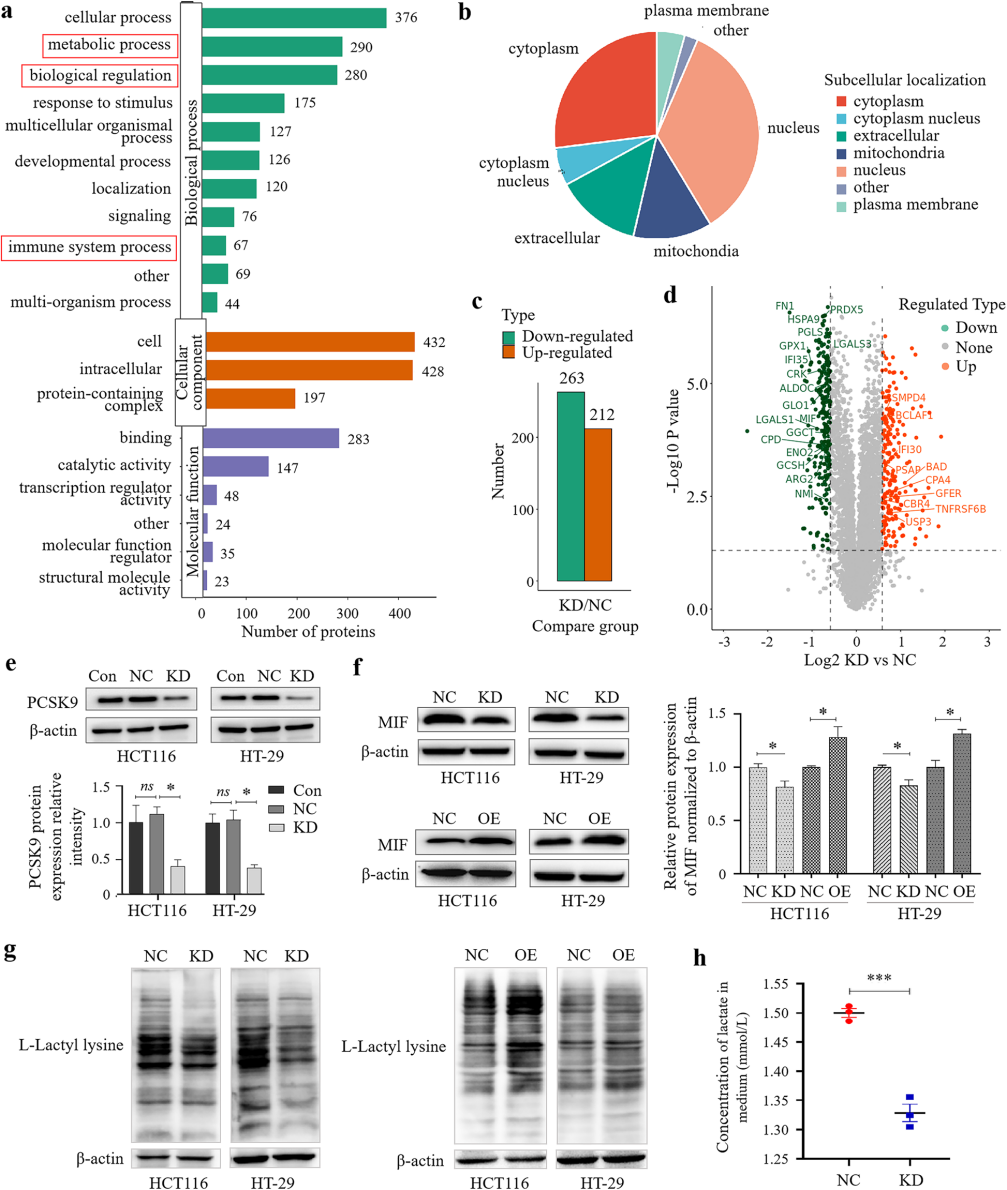
Proteomic analysis of differentially expressed proteins in colon cancer cells. **a**, The 4D-label-free quantitative proteomics by the gene ontology (GO) enrichment analysis. **b**, Subcellular localization of these differentially expressed proteins. **c**, The analysis identified statistics of differentially expressed proteins after PCSK9 knockdown in HCT116 cells. **d**, The volcano plot showed differential proteins associated with glycometabolism and immune reactions. **e **and** f**, Western blot. HCT116 and HT-29 cells were grown and transiently transfected with PCSK9 or control siRNA or with plasmids carrying PCSK9 cDNA or vector only and then subjected to Western blot analysis at 48 hs after transfaction. **g**, Western blot. HCT116 and HT-29 cells were grown and transiently transfected with PCSK9 or control siRNA or with plasmids carrying PCSK9 cDNA or vector only and then subjected to Western blot analysis of levels of protein lactylation. **h**, Concentration of lactate in the culture supernatant of HCT116 cells was established by Lactate Assay Kit. NC, negative control; KD, knockdown of PCSK9 cells transfected with PCSK9 siRNA; OE, overexpression of PCSK9 cells transfected with plasmids carrying PCSK9 cDNA. *p < 0.05; ***p < 0.001

To further explore the underlying molecular mechanisms of PCSK9 action on colon cancer progression, we performed the 4D-label-free quantitative proteomic analysis using the criteria of *p* ≤ 0.05 and the fold-change cutoff of quantitative protein rations more than 1.5 or less than 1/1.5 were considered statistically significant (Fig. 6a-d). We performed the gene ontology (GO) enrichment analysis of the main functions of these differentially expressed proteins. Our data showed that these differentially expressed proteins were mainly enriched in the cellular and metabolic process or immune system process (Fig. 6a). These differentially expressed proteins were mostly distributed in the nucleus, followed by the cytoplasm (Fig. 6b).

Subsequently, we analyzed the data using PCSK9-knocked down HCT116 cells (Fig. 6e) and found that a total of 475 proteins had a significant change, including 212 upregulated proteins and 263 downregulated proteins (Fig. 6c). We then performed the Kyoto Encyclopedia of Genes and Genomes (KEGG) database analysis and found that functions of these differentially expressed proteins were closely related to the cellular processes, immune reactions, signaling transduction and metabolism. The resulting volcano plot showed differential proteins associated with glycometabolism and immune reactions, some of which were showed in Fig. 6d. From these data, we found that the downregulation ratio (the KD/NC ratio) of macrophage migration inhibitory factor (MIF) is 0.6344 (*p* value = 0.00025) and more importantly, MIF has been known to be involved in EMT process, colorectal cancer progression and metastasis and phenotypic polarization of macrophages [30–32, 35–38].

According to these results, we performed Western blot to confirm expression of MIF proteins after knockdown of PCSK9 in HCT116 and HT-29 cells and found that PCSK9 knockdown in colon cancer cells significantly decreased MIF expression, consistent with the results of our proteomic analysis. In contrast, overexpression of PCSK9 significantly increased MIF protein levels in colon cancer cells (Fig. 6f). Then, we explored PCSK9 regulation of the levels of protein lactylation by performed Western blot analysis using an anti-L-Lactyl lysine antibody and detected the levels of lactate in the cell supernatant of colon cancer cells by Lactate Assay Kit. Our data showed that levels of the lactate-modified proteins were significantly reduced in HCT116 and HT-29 cells after knockdown of PCSK9 expression, whereas PCSK9 overexpression upregulated levels of the lactate-modified proteins in HCT116 and HT-29 cells (Fig. 6g). Besides, the concentration of lactate in the culture supernatant of HCT116 cells was significantly reduced after PCSK9 knockdown and thus we can deduce that PCSK9 promotes the level of lactate and protein lactylation in colon cancer cells (Fig. 6h).

### Inhibition of M2 macrophage polarization (but promotion of M1 polarization) co-cultured with colon cancer cells after knockdown of PCSK9 expression

**Fig. 7:**
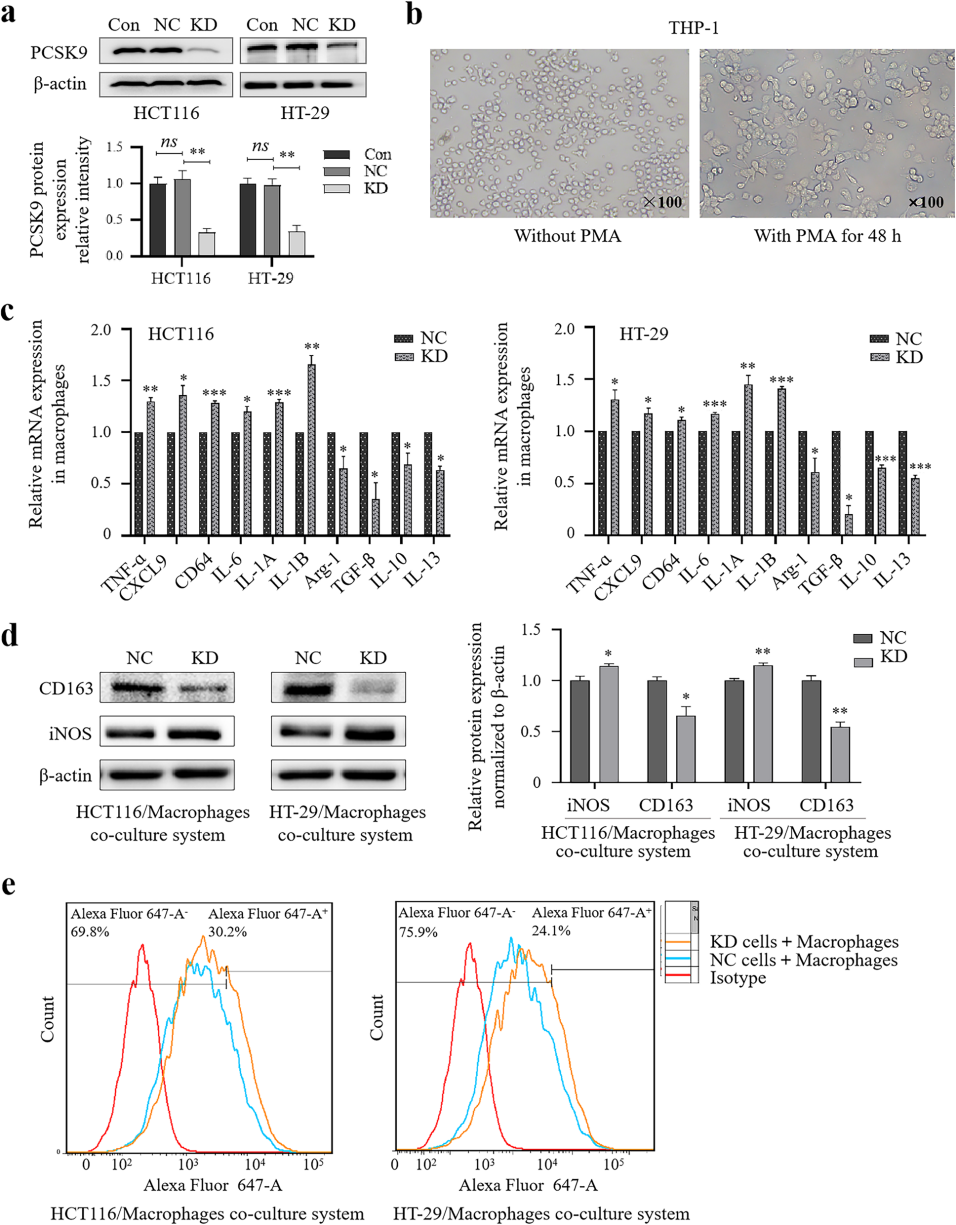
Inhibition of macrophage activation after co-culture with colon cancer cells. **a**, Quantitation data based on the mean levels of western blots at 48 hs after transfaction (n = 3). **b**, Cell morphology of macrophages. THP-1 cells were grown and treated with 100 ng/ml of phorbol 12-myristate 13-acetate (PMA) for 48 h. **c**, qRT-PCR. The co-cultured THP-1 cells were analyzed for mRNA levels of the M1 or M2 macrophages markers using qRT-PCR. **d**, Western blot. The co-cultured THP-1 cells were analyzed for CD163 and iNOS proteins using Western blot. **e**, Flow cytometry. The co-cultured THP-1 cells were analyzed for CD86^+^ macrophages. NC, negative control cells transfected with negative control siRNA; KD, knockdown of PCSK9 cells transfected with PCSK9 siRNA. *p < 0.05; **p < 0.01; ***p < 0.001

Tumor-associated macrophage (TAMs), as a part of the tumor microenvironment, are mainly derived from blood monocytes [39]. Functionally, TAMs can be classified into classically activated or inflammatory (M1) and alternatively activated or anti-inflammatory (M2) macrophages [40]. In other words, M1 macrophages inhibit tumor growth, whereas M2 macrophages promote tumor growth. Thus, inhibition of M2 macrophages or repolarization of M2 macrophages to M1 macrophages is common strategies to treat solid tumors [40], while importantly, lactate and MIF induce the polarization of TAMs [35]. In this regard, our data showed that PCSK9 regulated MIF expression and lactate and protein lactylation levels in colon cancer cells (Fig. 6); thus, we co-cultured HCT116 or HT-29 cells after PCSK9 knockdown (Fig. 7a) with THP-1-derived macrophages using the Transwell system and detected the expression of the M1/M2 markers using qRT-PCR, Western blot, and Flow cytometry assay.

We found that THP-1 cells were induced from suspension to adherent growth after incubation with PMA, and the cells stopped proliferating, changing from a round to spindle or irregular shape (Fig. 7b). At gene levels, qRT-PCR data demonstrated that compared with NC group, levels of M1 marker IL-6, IL-1A, IL-1B, CXCL9, CD64 and TNF-α mRNA were upregulated, whereas levels of M2 markers IL-10, IL-13, Arg-1, and TGF-β mRNA were downregulated in siPCSK9 group of cells (Fig. 7c). Western blot data revealed that expression of iNOS protein was induced, whereas CD163 protein was reduced in the siPCSK9 group of cells compared with the level in NC cells (Fig. 7d). Moreover, Flow cytometric analysis of CD86^+^ macrophages showed that knockdown of PCSK9 expression in HCT116 and HT-29 cells increased the numbers of CD86 + macrophages (Fig. 7e), indicating that knockdown of PCSK9 increased in M1 macrophages.

### Inhibition of colon cancer cell metastasis after knockdown of PCSK9 expression in vivo

**Fig. 8:**
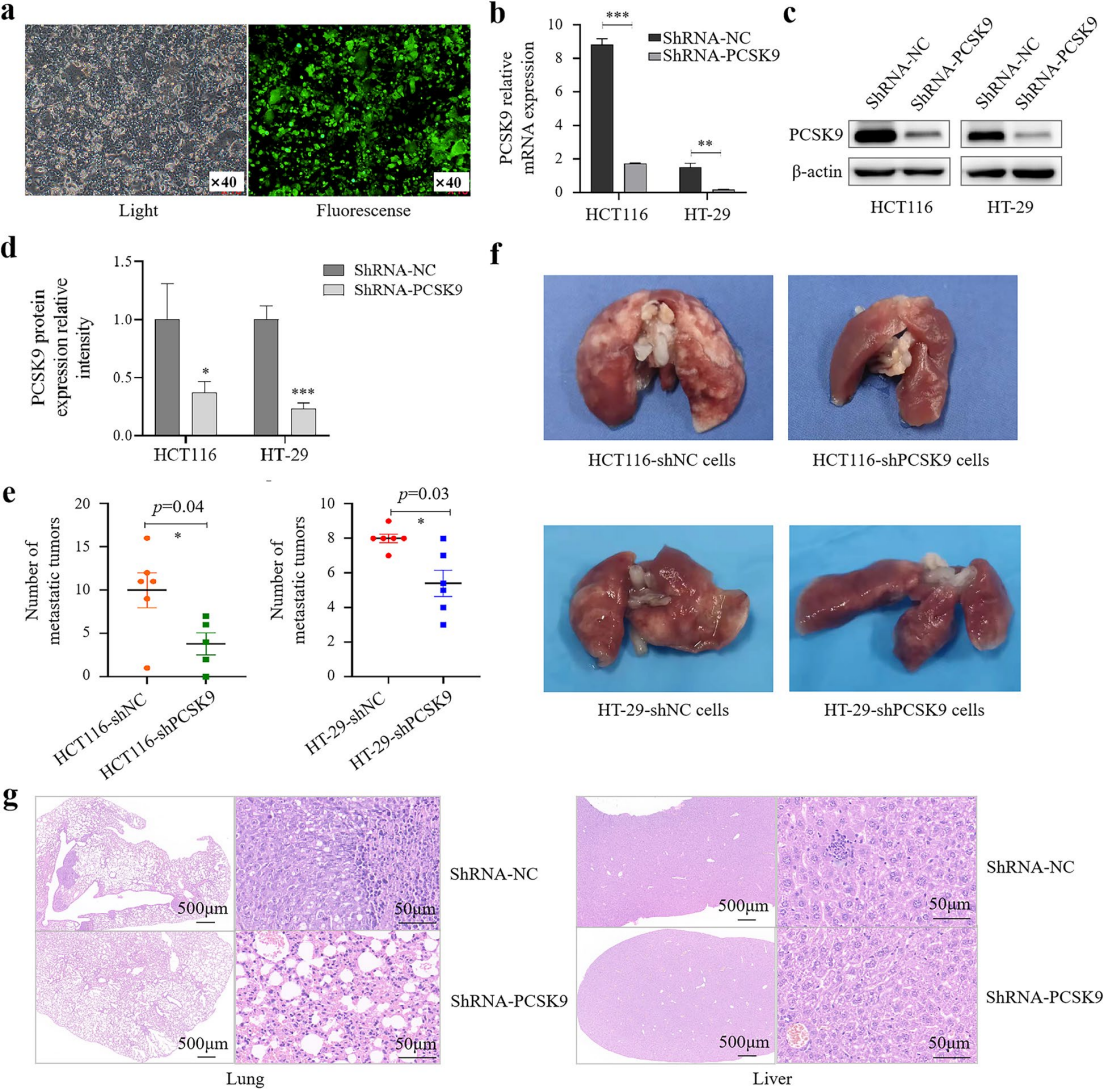
Inhibition of colon cancer cell metastasis after knockdown of PCSK9 expression in vivo. **a**, Fluorescence microscopy. HCT116 and HT-29 cells were grown and infected with lentivirus carrying PCSK9 siRNA or negative control siRNA for 48 h, and then subjected to fluorescence microscopy. **b–d**, qRT-PCR and Western blot. The assays confirmed the PCSK9 protein knockdown in tumor cells. **e**, Nude mouse xenograft model. Morphology of lung metastasis in PCSK9 deficiency HCT116 cells. **f**, The tail vein-lung metastasis model. Cells were injected into the tail vein to produce tumor cell lung metastasis. **g**, H&E staining. Lung and liver tissues were resected at the end of experiment and processed for tissue section and H&E staining to show histopathological changes of the lung and liver. Scale bar, 500 µm (a low magnification) or 50 µm (a high magnification). NC, negative control; *p < 0.05; **p < 0.01; ***p < 0.001

We first generated stably knocked down HCT116 and HT-29 cells and performed fluorescent microscopy to show successful infection of lentivirus carrying PCSK9 siRNA or negative control siRNA into tumor cells (Fig. 8a), while qRT-PCR and Western blot confirmed PCSK9 expression in shRNA-PCSK9 cells versus shRNA-NC cells (Fig. 8b–d). After that, we subcutaneously injected tumor cells into nude mice to produce a mouse xenograft tumor model.

After 6 weeks, there was no significant difference in size of the tumor cell xenografts between the NC and the PCSK9-silencing cell injections. During the experiment, one mouse with injection of PCSK9-silencing tumor cells died and the necropsy showed no tumor liver and lung metastasis. Moreover, liver metastasis was rare in all mice and there was no significant difference in tumor liver metastasis between these two groups at the end point of experiments; however, we found lung metastasis in all six mice with NC cell injection, but in mice with PCSK9-silencing cell injections, while lung metastasis of NC cell group was significantly more serious than that in PCSK9-silencing cell group (Fig. 8e).

Furthermore, we produced an in vivo tail vein-lung metastasis model by injecting PCSK9-silencing HT-29 cells into BALB/C nude mice, and 5 weeks later, the mice showed obvious emaciation and were sacrificed for analysis of tumor cell lung metastasis. We found that all 12 mice in both NC and PCSK9 silencing groups developed the rough lung surface, with translucent scattered or fragmented metastatic nodules. But the number of metastasis nodules in lung tissues was lower in shRNA-PCSK9 cell group than in shRNA-NC cell group (Fig. 8f). In addition, H&E staining showed slightly thickening of the alveolar walls in small areas with accompanied by a small number of granulocytes, and small focal mild hemorrhage in lung tissues of shRNA-PCSK9 cell-injected mice, while all bronchi were intact without obvious abnormalities (Fig. 8g). However, in lung tissues of shRNA-NC cell-injected mice, there was a visible lymphocyte necrosis in the lymph nodes around the bronchus, occasional cavitation, small area of mild thickening of the alveolar wall, visible mononuclear cells and granulocyte infiltration with a stove mild bleeding, a small amount of eosinophilic material in bronchioles. Tumor cells of lung metastases were mostly scattered in the lung parenchyma, mainly around the bronchi, and some metastases were distributed around small blood vessels of the lung. Some lung tissue structures were destroyed, and alveoli disappeared in lung tissues of shRNA-NC cell-injected mice.

There was vascular congestion occasionally and degeneration of hepatocytes around the central vein and necrotic foci in hepatic tissues of the shRNA-NC cell-injected mice, whereas there was a mild degeneration of hepatocytes without obvious inflammatory cell infiltration and necrotic foci, but there was only a small amount of central venous congestion in shRNA-PCSK9 cell-injected mice (Fig. 8g). Liver metastasis was rare in all mice. Collectly, knockdown of PCSK9 expression could attenuate colon cancer cell lung metastasis in vivo.

## Discussion

Potential mechanisms contributing to increased cholesterol availability in cancer cell growth are believed to include tumor cells utilized cholesterol, uptake from the blood or de novo synthesis for membrane and lipid raft biosynthesis, and signaling molecules to facilitate their fast growth needs [41], while PCSK9 binds to the LDL-R to regulate lipoprotein homeostasis [8, 9] and immune checkpoints in cancer [42]. Indeed, altered cholesterol synthesis pathway did play an important role in tumorigenesis since recent TCGA project and preclinical data provided evidence in support of the cholesterol pathway in cancer development [43]. Molecular studies demonstrated a cholesterol accumulation or high cholesterol content in tumor tissues of breast, thyroid, uterine, ovarian, and renal cancers versus their normal tissues [44]. Numerous pre-clinical, clinical, meta-analysis, and population-based studies revealed associations of cholesterol level with cancer risk, tumor progression, poor survival, or prognosis and fewer responses to therapy in different human cancers [44–47]. However, in colon cancer, there was inverse association between high blood cholesterol and a risk in developing colon cancer [48], or there is no protective effect of the elevated blood cholesterol level on colon cancer development and progression [48]. A previous pre-clinical study reported that LDL-C was able to enhance intestinal inflammation and colon cancer progression by activation of the ROS and MAPK signaling [48]. There was also a difference in gender between cholesterol and colon cancer risk [49]. Recent researched have confirmed the anti-tumor effects of PCSK9 inhibition [50–52]. However, PCSK9 knockout substantially suppressed tumor cell growth in mice depending on cytotoxic T cells and the inhibitory effects of PCSK9 was independent of its hypolipidemic pathway [24], which thus promoted us to further explore the role of PCSK9 in colon cancer.

Our current study showed that PCSK9 expression was upregulated in colon cancer tissues and associated with advanced tumor pathological grade, while the reduction of PCSK9 expression affected colon cancer cell proliferation, migration, and invasion in vitro. Moreover, knockdown of PCSK9 expression reduced colon cancer cell EMT and the activation of the PI3K/AKT signaling, while our proteomic analysis showed that the reduction of PCSK9 expression affected colon cancer cell metabolism as well as immune reactions to induce M1 macrophage polarization. PCSK9 played an important role in the progression and metastasis of colon cancer by regulation of tumor cell EMT and PI3K/AKT signaling and in the phenotypic polarization of macrophages by mediating MIF and lactate levels.

The data from our current study revealed that the influence of PCSK9 protein on different cellular processes in colon cancer and targeting of PCSK9 could be a novel approach to control of colon cancer in future clinical practice. Indeed, a previous animal study demonstrated that PCSK9 inhibition using the nano-liposomal anti-PCSK9 vaccine had a moderately inhibitory activity in tumor growth and improved the lifespan and survival of the mice, thus providing support for the potential use of PCSK9 inhibitors in the treatment of colon cancer [53]. Our previous in vivo study also revealed that PCSK9 expression was able to promote growth and malignant transformation of intestinal tumors in mice with *Apc* mutation [54].

Our study demonstrated that PCSK9 promotes the progression and metastasis of colon cancer cells through multiple pathways. Indeed, during cell EMT, the cells lost the apicobasal polarity and intercellular junctions, while expression of the epithelial markers (such as E-cadherin) was downregulated. In contrast, the mesenchymal phenotypes and expression of related markers (such as N-cadherin or vimentin) are induced, leading to changes in cell biological behaviors, like enhancement of cell migration and invasion ability [30, 31]. Thus, tumor cell EMT is closely related to cancer invasion and metastasis and plays a crucial role in distant metastasis [31, 32]. The molecular mechanisms of the EMT activation are complex, involving various molecules and signaling pathways [32]; for example, various transcription factors, including the ZEB family (ZEB1 and ZEB2), Snail family (Snail 1, 2, and 3), and Twist family (Twist 1 and 2) [32] could regulate E-cadherin gene transcription [32]. In addition, the matrix metalloproteinases (MMPs) can degrade the extracellular matrix components to promote cancer cell invasion and metastasis [30]. Expression of MMPs, especially MMP-2 and MMP-9, was regulated by Snail 1 [30].

Furthermore, the MIF, a pro-inflammatory cytokine, plays an important role in regulation of the innate and adaptive immune responses, alteration of which was associated with development of different human cancers; for example, the MIF signaling pathway was able to upregulate expression of various cancer-promoting genes, which can not only act directly on tumors, but also promote tumorigenesis by acting on tumor microenvironment [36, 37]. Previous studies demonstrated that MIF was able to induce macrophage M2 polarization, while knockdown of MIF expression facilitated macrophage M1 polarization in a various types of cancers [35–38, 55]. As we know, the metabolic reprogramming is one of the hallmarks in cancer cells, one of which is to increase glucose uptake and produce more lactate, even under aerobic conditions [56]. As a signaling molecule, lactate in the tumor microenvironment plays a special role in tumor proliferation, metastasis, angiogenesis, macrophage polarization, immune escape, and chemotherapy resistance [57]. Zhang et al. for the first time revealed the epigenetic regulation mechanism of histone lysine lactylation, and its role in gene expression and modification of macrophages [58]. The lysine lactylation level was associated with increased concentration of endogenous lactate [58]. Furthermore, lactate and lysine lactylation had an effect on macrophage functions and played an important role in expression of M2-related genes during macrophage M1 polarization [58], metabolic properties and preferences during cancer progression, therapy resistance, and metastasis [59]. Tumor lactate levels were associated with an increase in cancer metastasis, recurrence, and poor outcomes [60]. Lactate also plays role in promoting tumor progression and in functioning as a signaling molecule that stimulates TAMs polarization and tumor metastasis [56–60].

We utilized the proteomic analysis to demonstrate that the reduction of PCSK9 expression in colon cancer cells induced differentially expressed proteins that were mainly enriched in cellular and metabolic processes, while level of MIF expression had a significant trend for decline. We also found that levels of lactate and protein lactylation was reduced in colon cancer cells after the reduction of PCSK9 expression. In contrast, levels of lactate, protein lactylation and MIF protein were significantly enhanced in colon cancer cells after PCSK9 overexpression. Our data suggest that levels of protein lactylation were associated with exogenous and endogenous lactate. Thus, we speculate that PCSK9 could be involved in regulation of the intracellular lactate metabolism in colon cancer cells. Considering that both lactate and MIF regulate antitumor immunity and play a role in tumor microenvironment, we analyzed PCSK9 in regulation of TAMs polarization and found that PCSK9 was able to alter TAMs polarization in order to change the functions of TAMs in colon cancer. Moreover, macrophage phenotypic polarization induced by PCSK9 might be attributed to its regulatory function on the levels of MIF and lactate.

## Conclusions

**Fig. 9:**
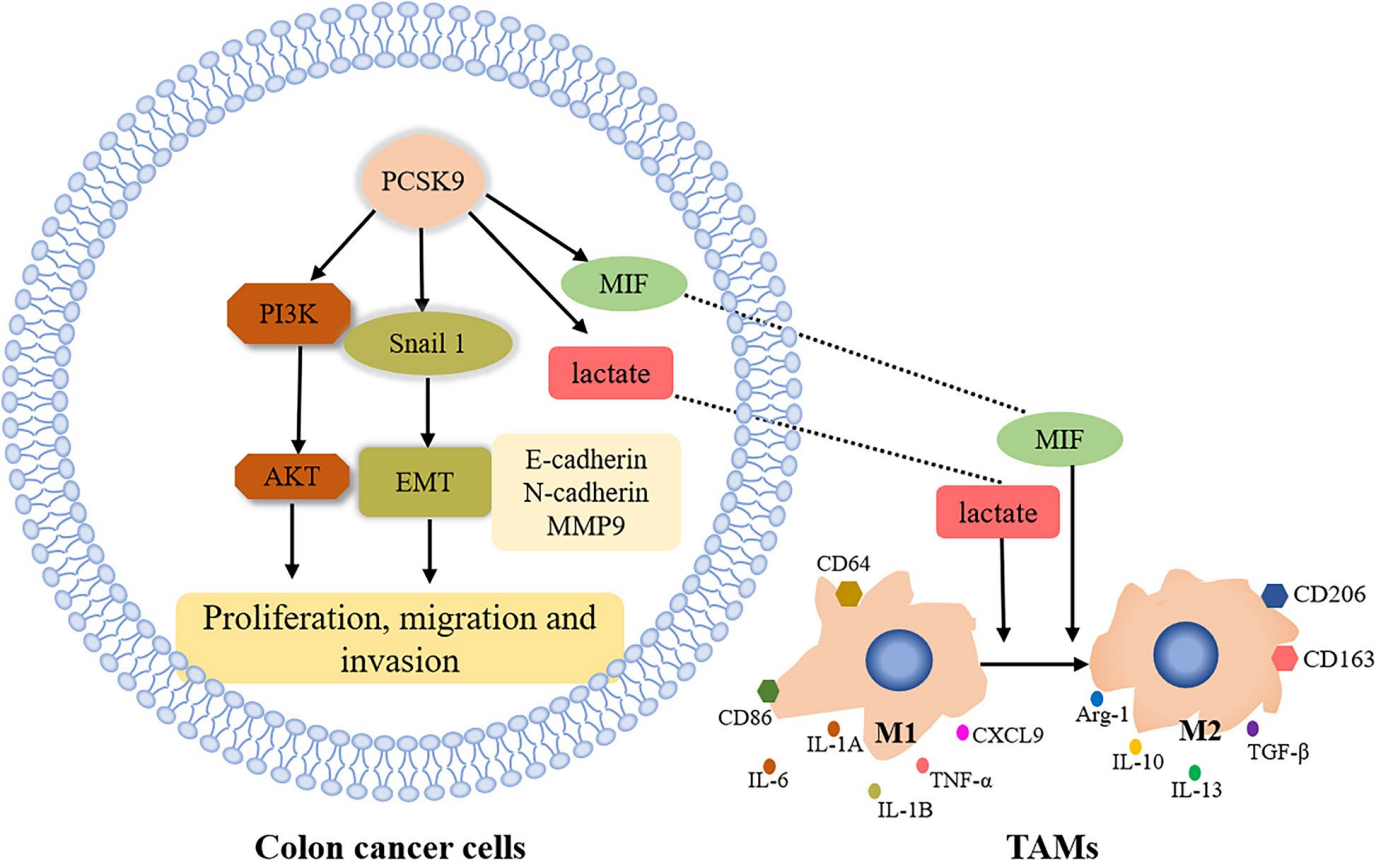
Illustration of PCSK9 oncogenic activity in colon cancer. PCSK9 protein promotes colon cancer cell proliferation and metastasis by activation of the PI3K/AKT pathway and tumor cell EMT. PCSK9 also induces M2 macrophage polarization might through changes in lactate and MIF levels; thus, PCSK9 inhibition could reverse this trend; thereby enhancing human body antitumor immunity

In summary, our current study comprehensively assessed the role of PCSK9 in progression and metastasis of colon cancer by analysis of colon cancer tissue samples, in vitro cell experiments, and in vivo animal experiments. We revealed that PCSK9 protein was highly expressed in colon cancer tissues and associated with advanced tumor pathological grade. Moreover, PCSK9 expression enhanced colon cancer cell proliferation, migration, and invasion in vitro by induction of tumor cell EMT and activation of the PI3K/AKT signaling. Besides, PCSK9 directly or indirectly activated Snail 1 and subsequently to downregulate E-cadherin (but upregulate N-cadherin and MMP9) and then induce the colon cancer cell EMT process. Furthermore, PCSK9 promoted M2 macrophage polarization, but it inhibited M1 macrophage polarization by mediating MIF and lactate levels (Fig. 9). Collectively, our current data demonstrated the oncogenic activity of PCSK9 in colon cancer. A future study will investigate PCSK9 inhibition as a potential therapeutic approach to control colon cancer clinically.

## Supplementary Information

**Additional file 1:** **Supplemental Table S1. **Colon cancer tissue samples information

**Additional file 2:** **Supplemental Table S2. **ThesiRNA sequences of NC and siPCSK9

**Additional file 3:** **Supplemental Table S3.** Plasmid sequence of PCSK9

**Additional file 4:** **Supplemental Table S4.** Primers used for qRT- PCR

**Additional file 5.** 

**Additional file 6.** 

## Data Availability

All data generated or analyzed during this study are included in this article and its supplementary files.

## Abbreviations

PCSK9: Proprotein convertase subtilisin/kexin type 9
LDL-R: Low-density lipoprotein receptor
NARC-1: Neural apoptosis-regulated convertase 1
EMT: Epithelial–mesenchymal transition
TMA: Tissue microarray
TCGA: The Cancer Genome Atlas
COAD: Colon adenocarcinoma
MOI: Multiplicities of infection
CCK8: Cell Counting Kit-8
OD450: Optical density value
PBS: Phosphate buffered saline
HPLC: High-performance liquid chromatography
LC–MS: Liquid chromatography-mass spectrometry
PSM: Peptide spectral matching
BCA: Bicinchoninic acid
SDS-PAGE: Sodium dodecyl sulfate–polyacrylamide gel electrophoresis
PVDF: Polyvinylidene fluoride; enhanced chemiluminescence
HRP: Horseradish peroxidase
ECL: Enhanced chemiluminescence
ANOVAL: One-way analysis of variance
PI3K/AKT: Phosphatidylinositol 3-kinase/Protein Kinase-B
GO: Gene ontology
KEGG: Kyoto Encyclopedia of Genes and Genomes
MIF: Migration inhibitory factor
TAMs: Tumor-associated macrophages
MMPs: Matrix metalloproteinases
