# Supplementary material for: PCSK9 promotes the progression and metastasis of colon cancer cells through regulation of EMT and PI3K/AKT signaling in tumor cells and phenotypic polarization of macrophages
Source: J Exp Clin Cancer Res. 2022 Oct 14;41:303. doi: 10.1186/s13046-022-02477-0 (PMC9563506; doi:10.1186/s13046-022-02477-0)
Supplement: Supplementary file 1 — Additional file 1: Supplemental Table S1. Colon cancer tissue samples information [file 13046_2022_2477_MOESM1_ESM.doc]

**Supplementary Information**

**Supplemental Table S1. Colon cancer tissue samples information.**

| Tissue type | Tissue coding | Tumor type | Sex | Age | Distant site of metastasis | Primary organ | Pathological classification | Pathological grade | Tumor size (cm) | Tumor site | N | M | Clinical staging |
| --- | --- | --- | --- | --- | --- | --- | --- | --- | --- | --- | --- | --- | --- |
| Carcinoma/Paracancer/Distal | D15A1968 | Colon | M | 59 | No | Yes | Adenocarcinoma | I | φ5 | Colon | N0 | M0 | 1-2 |
| Carcinoma/Paracancer/Distal | D15A1986 | Colon | F | 65 | No | Yes | Adenocarcinoma | I | φ3 | Sigmoid | N0 | M0 | 1-2 |
| Carcinoma/Paracancer/Distal | D15A1989 | Colon | M | 49 | No | Yes | Adenocarcinoma | Ⅰ-Ⅲ | φ3 | Transverse colon | N0 | M0 | 1-2 |
| Carcinoma/Paracancer/Distal | D15A1942 | Colon | F | 28 | No | Yes | Adenocarcinoma | Ⅱ | φ4 | Ileocecal colon | N0 | M0 | 1-2 |
| Carcinoma/Paracancer/Distal | D15A1975 | Colon | F | 68 | No | Yes | Adenocarcinoma | Ⅱ | φ3.5 | Transverse colon | N0 | M0 | 1-2 |
| Carcinoma/Paracancer/Distal | D15A1977 | Colon | M | 70 | No | Yes | Adenocarcinoma | Ⅱ | φ4.5 | Sigmoid | N0 | M0 | 1-2 |
| Carcinoma/Paracancer/Distal | D15A1979 | Colon | M | 54 | No | Yes | Adenocarcinoma | Ⅱ | φ3 | Ascending colon | N0 | M0 | 1-2 |
| Carcinoma/Paracancer/Distal | D15A1982 | Colon | M | 74 | No | Yes | Adenocarcinoma | Ⅱ | φ2.5 | Transverse colon | N0 | M0 | 1-2 |
| Carcinoma/Paracancer/Distal | D15A1945 | Colon | F | 54 | No | Yes | Adenocarcinoma | Ⅱ-Ⅲ | φ3 | Transverse colon | N0 | M0 | 1-2 |
| Carcinoma/Paracancer/Distal | D15A1962 | Colon | F | 57 | No | Yes | Adenocarcinoma | Ⅱ-Ⅲ | φ4 | Ascending colon | N0 | M0 | 1-2 |
| Carcinoma/Paracancer/Distal | D15A1969 | Colon | M | 60 | No | Yes | Adenocarcinoma | Ⅲ | φ4 | Sigmoid | N0 | M0 | 1-2 |
| Carcinoma/Paracancer/Distal | D15A1978 | Colon | M | 59 | No | Yes | Adenocarcinoma | Ⅰ-Ⅱ | φ4 | Transverse colon | L | M0 | 3 |
| Carcinoma/Paracancer/Distal | D15A1984 | Colon | M | 56 | No | Yes | Adenocarcinoma | Ⅰ-Ⅱ | φ5 | Sigmoid | L | M0 | 3 |
| Carcinoma/Paracancer/Distal | D15A1958 | Colon | M | 50 | No | Yes | Adenocarcinoma | Ⅰ-Ⅱ | φ4.5 | Sigmoid | L | M0 | 3 |
| Carcinoma/Paracancer/Distal | D15A1941 | Colon | M | 51 | No | Yes | Adenocarcinoma | Ⅱ | φ5.0 | Transverse colon | L | M0 | 3 |
| Carcinoma/Paracancer/Distal | D15A1946 | Colon | M | 61 | No | Yes | Adenocarcinoma | Ⅱ | φ4.5 | Ascending colon | L | M0 | 3 |
| Carcinoma/Paracancer/Distal | D15A1951 | Colon | M | 27 | No | Yes | Adenocarcinoma | Ⅱ | φ3.5 | Colon | L | M0 | 3 |
| Carcinoma/Paracancer/Distal | D15A1956 | Colon | F | 62 | No | Yes | Adenocarcinoma | Ⅱ | φ4.5 | Descending colon | L | M0 | 3 |
| Carcinoma/Paracancer/Distal | D15A1960 | Colon | M | 49 | No | Yes | Adenocarcinoma | Ⅱ | φ4.5 | Transverse colon | L | M0 | 3 |
| Carcinoma/Paracancer/Distal | D15A1961 | Colon | M | 52 | No | Yes | Adenocarcinoma | Ⅱ | φ5 | Ascending colon | L | M0 | 3 |
| Carcinoma/Paracancer/Distal | D15A1964 | Colon | F | 47 | No | Yes | Adenocarcinoma | Ⅱ | φ4 | Sigmoid | L | M0 | 3 |
| Carcinoma/Paracancer/Distal | D15A1970 | Colon | M | 44 | No | Yes | Adenocarcinoma | Ⅱ | φ4.5 | Right hemicolon | L | M0 | 3 |
| Carcinoma/Paracancer/Distal | D15A1972 | Colon | M | 53 | No | Yes | Adenocarcinoma | Ⅱ | φ3.5 | Transverse colon | L | M0 | 3 |
| Carcinoma/Paracancer/Distal | D15A1980 | Colon | F | 58 | No | Yes | Adenocarcinoma | Ⅱ | φ5 | Right colon | L | M0 | 3 |
| Carcinoma/Paracancer/Distal | D15A1981 | Colon | M | 53 | No | Yes | Adenocarcinoma | Ⅱ | φ4.5 | Right hemicolon | L | M0 | 3 |
| Carcinoma/Paracancer/Distal | D15A1983 | Colon | M | 53 | No | Yes | Adenocarcinoma | Ⅱ | φ4 | Right hemicolon | L | M0 | 3 |
| Carcinoma/Paracancer/Distal | D15A1988 | Colon | M | 39 | No | Yes | Adenocarcinoma | Ⅱ | φ3.5 | Sigmoid | L | M0 | 3 |
| Carcinoma/Paracancer/Distal | D15A1938 | Colon | M | 73 | No | Yes | Adenocarcinoma | Ⅱ-Ⅲ | φ4.5 | Transverse colon | L | M0 | 3 |
| Carcinoma/Paracancer/Distal | D15A1971 | Colon | F | 51 | No | Yes | Adenocarcinoma | Ⅱ-Ⅲ | φ6.5 | Descending colon | L | M0 | 3 |
| Carcinoma/Paracancer/Distal | D15A1974 | Colon | F | 86 | No | Yes | Adenocarcinoma | Ⅲ | φ5.5 | Ascending colon | L | M0 | 3 |

Note: M, male; F, female; L, Lymph node metastasis
