# Supplementary material for: PCSK9 promotes the progression and metastasis of colon cancer cells through regulation of EMT and PI3K/AKT signaling in tumor cells and phenotypic polarization of macrophages
Source: J Exp Clin Cancer Res. 2022 Oct 14;41:303. doi: 10.1186/s13046-022-02477-0 (PMC9563506; doi:10.1186/s13046-022-02477-0)
Supplement: Supplementary file 2 — Additional file 2: Supplemental Table S2. ThesiRNA sequences of NC and siPCSK9 [file 13046_2022_2477_MOESM2_ESM.doc]

***Supplemental Table S2*** *The* *siRNA sequences of NC and siPCSK9*

|  | Sence (5’-3’) | Anti-sense (5’-3’) |
| --- | --- | --- |
| siRNA-NC | UUCUCCGAACGUGUCACGUTT | ACGUGACACGUUCGGAGAATT |
| siRNA-PCSK9 | GGGUCAUGGUCACCGACUUCG | AAGUCGGUGACCAUGACCCUG |
