# Supplementary material for: PCSK9 promotes the progression and metastasis of colon cancer cells through regulation of EMT and PI3K/AKT signaling in tumor cells and phenotypic polarization of macrophages
Source: J Exp Clin Cancer Res. 2022 Oct 14;41:303. doi: 10.1186/s13046-022-02477-0 (PMC9563506; doi:10.1186/s13046-022-02477-0)
Supplement: Supplementary file 3 — Additional file 3: Supplemental Table S3. Plasmid sequence of PCSK9 [file 13046_2022_2477_MOESM3_ESM.doc]

**Supplemental Table S3** Plasmid sequence of PCSK9

|  | Plasmid sequence |
| --- | --- |
| Vector (pEX-3) | TACAGTGGGAGGTGGGAGTTTTTTAAGCAAGTAAAACCTCTACAAATGTGGTATGGCTGATTATGATCTAGAGTCGCGGCCGCATTGATATCAATGGCGCGCCGATACCGGTGATGGATCCCGGGCCCGCGGTACCGTCGACTGCAGAATTCTCACTGGAGCTCCTGGGAGGCCTGCGCCAGGTGCCGGCTCCGGCAGCAGATGGCAACGGCTGTCACGGCCCCTTCGCTGGTGCTGCCTGTAGTGCTGACGTCCCGGCTCCTGACTACACACGTGTTGTCTACGGCGTAGGCCCCCAGGACGTGGGAGGTCCCAGGGAGGGCACTGCAGCCAGTCAGGGTCCAGCCCTCCTCGCAGGCCACGGTCACCTGCTCCTGAGGGGCCGGGATTCCATGCTCCTTGACTTTGCATTCCAGACCTGGGGCATGGCAGCAGGAAGCGTGGATGCTGGCCTCCCTGTGGCCCACGCACTGGTTGGGCTGACCTCGTGGCCTCAGCACAGGCGGCTTGTGGGTGCCAAGGTCCTCCACCTCCCAGTGGGAGCTGCAGCCTGTGAGGACGTGGCCCTGTTGGTGGCAGTGGACACGGGTCCCCATGCTGGCCTCAGCTGGTGGAGCTGTGTGGACGCTGCAGTTGGCCTGGGGTAGCAGGCAGCACCTGGCAATGGCGTAGACACCCTCACCCCCAAAAGCGTTGTGGGCCCGGCAGACCAGCTTGCCCCCTTGGGCCTCCATGCGCTCGCCCCGCCGCTTCCCACTCCTGGAGAAACTGGAGCAGCTCAGCAGCTCCTCATCTGGGGCGCAGCGGGCGACGGCTGTGGCCATCCGTGTAGGCCCCGAGTGTGCTGACCATACAGTCCTGCAAAACAGCTGCCAACCTGCCCCATGGGTGCTGGGGGGCAGGGCGGCCACCAGGTTGGGGGTCAGTACCCGCTGGTCCTCAGGGAACCAGGCCTCATTGATGACATCTTTGGCAGAGAAGTGGATCAGTCTCTGCCTCA |
| PCSK9 (Human) | ATGGGCACCGTCAGCTCCAGGCGGTCCTGGTGGCCGCTGCCACTGCTGCTGCTGCTGCTGCTGCTCCTGGGTCCCGCGGGCGCCCGTGCGCAGGAGGACGAGGACGGCGACTACGAGGAGCTGGTGCTAGCCTTGCGTTCCGAGGAGGACGGCCTGGCCGAAGCACCCGAGCACGGAACCACAGCCACCTTCCACCGCTGCGCCAAGGATCCGTGGAGGTTGCCTGGCACCTACGTGGTGGTGCTGAAGGAGGAGACCCACCTCTCGCAGTCAGAGCGCACTGCCCGCCGCCTGCAGGCCCAGGCTGCCCGCCGGGGATACCTCACCAAGATCCTGCATGTCTTCCATGGCCTTCTTCCTGGCTTCCTGGTGAAGATGAGTGGCGACCTGCTGGAGCTGGCCTTGAAGTTGCCCCATGTCGACTACATCGAGGAGGACTCCTCTGTCTTTGCCCAGAGCATCCCGTGGAACCTGGAGCGGATTACCCCTCCACGGTACCGGGCGGATGAATACCAGCCCCCCGACGGAGGCAGCCTGGTGGAGGTGTATCTCCTAGACACCAGCATACAGAGTGACCACCGGGAAATCGAGGGCAGGGTCATGGTCACCGACTTCGAGAATGTGCCCGAGGAGGACGGGACCCGCTTCCACAGACAGGCCAGCAAGTGTGACAGTCATGGCACCCACCTGGCAGGGGTGGTCAGCGGCCGGGATGCCGGCGTGGCCAAGGGTGCCAGCATGCGCAGCCTGCGCGTGCTCAACTGCCAAGGGAAGGGCACGGTTAGCGGCACCCTCATAGGCCTGGAGTTTATTCGGAAAAGCCAGCTGGTCCAGCCTGTGGGGCCACTGGTGGTGCTGCTGCCCCTGGCGGGTGGGTACAGCCGCGTCCTCAACGCCGCCTGCCAGCGCCTGGCGAGGGCTGGGGTCGTGCTGGTCACCGCTGCCGGCAACTTCCGGGACGATGCCTGCCTCTACTCCCCAGCCTCAGCTCCCGAGGTCATCACAGTTGGGGCCACCAATGCCCAAGACCAGCCGGTGACCCTGGGGACTTTGGGGACCAACTTTGGCCGCTGTGTGGACCTCTTTGCCCCAGGGGAGGACATCATTGGTGCCTCCAGCGACTGCAGCACCTGCTTTGTGTCACAGAGTGGGACATCACAGGCTGCTGCCCACGTGGCTGGCATTGCAGCCATGATGCTGTCTGCCGAGCCGGAGCTCACCCTGGCCGAGTTGAGGCAGAGACTGATCCACTTCTCTGCCAAAGATGTCATCAATGAGGCCTGGTTCCCTGAGGACCAGCGGGTACTGACCCCCAACCTGGTGGCCGCCCTGCCCCCCAGCACCCATGGGGCAGGTTGGCAGCTGTTTTGCAGGACTGTATGGTCAGCACACTCGGGGCCTACACGGATGGCCACAGCCGTCGCCCGCTGCGCCCCAGATGAGGAGCTGCTGAGCTGCTCCAGTTTCTCCAGGAGTGGGAAGCGGCGGGGCGAGCGCATGGAGGCCCAAGGGGGCAAGCTGGTCTGCCGGGCCCACAACGCTTTTGGGGGTGAGGGTGTCTACGCCATTGCCAGGTGCTGCCTGCTACCCCAGGCCAACTGCAGCGTCCACACAGCTCCACCAGCTGAGGCCAGCATGGGGACCCGTGTCCACTGCCACCAACAGGGCCACGTCCTCACAGGCTGCAGCTCCCACTGGGAGGTGGAGGACCTTGGCACCCACAAGCCGCCTGTGCTGAGGCCACGAGGTCAGCCCAACCAGTGCGTGGGCCACAGGGAGGCCAGCATCCACGCTTCCTGCTGCCATGCCCCAGGTCTGGAATGCAAAGTCAAGGAGCATGGAATCCCGGCCCCTCAGGAGCAGGTGACCGTGGCCTGCGAGGAGGGCTGGACCCTGACTGGCTGCAGTGCCCTCCCTGGGACCTCCCACGTCCTGGGGGCCTACGCCGTAGACAACACGTGTGTAGTCAGGAGCCGGGACGTCAGCACTACAGGCAGCACCAGCGAAGGGGCCGTGACAGCCGTTGCCATCTGCTGCCGGAGCCGGCACCTGGCGCAGGCCTCCCAGGAGCTCCAGTGA |
