# Supplementary material for: PCSK9 promotes the progression and metastasis of colon cancer cells through regulation of EMT and PI3K/AKT signaling in tumor cells and phenotypic polarization of macrophages
Source: J Exp Clin Cancer Res. 2022 Oct 14;41:303. doi: 10.1186/s13046-022-02477-0 (PMC9563506; doi:10.1186/s13046-022-02477-0)
Supplement: Supplementary file 4 — Additional file 4: Supplemental Table S4. Primers used for qRT- PCR [file 13046_2022_2477_MOESM4_ESM.doc]

**Supplemental Table S4** Primers used for qRT- PCR

| Genes | Forward | Reverse |
| --- | --- | --- |
| PCSK9 | CCTGGAGCGGATTACCCCT | CTGTATGCTGGTGTCTAGGAGA |
| IL-6 | ACTCACCTCTTCAGAACGAATTG | CCATCTTTGGAAGGTTCAGGTTG |
| IL-23 | CTCAGGGACAACAGTCAGTTC | ACAGGGCTATCAGGGAGCA |
| IL-1A | AGATGCCTGAGATACCCAAAACC | CCAAGCACACCCAGTAGTCT |
| IL-1B | AGCTACGAATCTCCGACCAC | CGTTATCCCATGTGTCGAAGAA |
| CXCL9 | CCAGTAGTGAGAAAGGGTCGC | AGGGCTTGGGGCAAATTGTT |
| CD64 | TGGCCTTGAGGTGTCATGC | GCAAGAGCAACTTTGTTTCACA |
| TNF-α | CCTCTCTCTAATCAGCCCTCTG | GAGGACCTGGGAGTAGATGAG |
| Arg-1 | TGGACAGACTAGGAATTGGCA | CCAGTCCGTCAACATCAAAACT |
| TGF-β | GGCCAGATCCTGTCCAAGC | GTGGGTTTCCACCATTAGCAC |
| IL-10 | TCAAGGCGCATGTGAACTCC | GATGTCAAACTCACTCATGGCT |
| IL-13 | GAGGATGCTGAGCGGATTCTG | CACCTCGATTTTGGTGTCTCG |
| GAPDH | GGGAGCCAAAAGGGTCATCA | TGATGGCATGGACTGTGGTC |
