# Supplementary material for: PCSK9 promotes the progression and metastasis of colon cancer cells through regulation of EMT and PI3K/AKT signaling in tumor cells and phenotypic polarization of macrophages
Source: J Exp Clin Cancer Res. 2022 Oct 14;41:303. doi: 10.1186/s13046-022-02477-0 (PMC9563506; doi:10.1186/s13046-022-02477-0)
Supplement: Supplementary file 5 — Additional file 5. [file 13046_2022_2477_MOESM5_ESM.pdf]

SHYJS-CP-1501006

	**上海芯超生物科技有限公司**<br>Shanghai Outdo Biotech Company	Barcode	
	伦理委员会审核意见书	编号/Ctl No.	YB M-05-02
		版本/Version	1.0

项目名称	结肠癌组织芯片产品开发及应用		
项目来源	上海芯超生物科技有限公司	项目承担单位	上海芯超生物科技有限公司
项目编号	HCol-Ade090PG-01	项目负责人	朱诗樟

审核结果

参加伦理委员会会议 7 人，投票结果：

A）同意 7 票
B）修正后再审查 ____ 票
C）不同意 ____ 票
D）暂停或终止 ____ 票

伦理委员会审查意见：

申报内容经审查，符合伦理要求，同意通过审查。

记录人签名：陈红[illegible]

2015年 1 月 9 日

主任委员签名：
（伦理委员会盖章）

上海芯超生物科技有限公司 伦理委员会

年 月 日

保存期限：长期

	上海芯超生物科技有限公司<br>Shanghai Outdo Biotech Company	Barcode	
	样本使用伦理审查申请表	编号/Ctl No.	YB M-05-01
		版本/Version	1.0

项目名称：	结肠癌组织芯片产品开发及应用	项目编号：	HCol-Ade090PG-01
项目来源：	上海芯超生物科技有限公司	资金来源：	上海芯超生物科技有限公司
样本名称：	结肠癌石蜡样本	样本数量：	癌 30 例/癌旁 30 例/远端 30 例
申请人：	沈晓静	联系方式：	021-51320288-5219
研究者 1：	高校、医院、研究所	研究者 2：	企业及其他研发机构
项目承担单位：	上海芯超生物科技有限公司		
项目负责人：	朱诗樟	联系方式：	021-51320288-5219
拟研究时间：	__2015_年__1__月_2_日 至 __2099__年__12__月_31___日		

一、 请阐述该样本用于研究的描述（包括目标和假设）：

结肠腺癌 30 例：癌/旁/远端。病理分级Ⅰ级Ⅱ级Ⅲ级。

二、项目研究的方法：免疫组化、原位杂交、原位 PCR 等分子病理学实验

备注	
	申报部门意见：<br><br>部门负责人签（章）：[signature]
说明	为有效利用宝贵的样本资源，您需向样本库提供一份简要的研究设计（如上）和所要求的资料，您要保证您将合法地使用样本，并进行您的上述研究，您需承诺在您的研究成果和文章上注明样本来源于：<br>上海芯超•生物样本库<br>申请程序：下载申请表----填写申请表----负责人签字（盖章）----样本库伦理委员会审核

申请人：[signature]<br><br>2015年 1 月 2 日	样本库审核意见：<br>样本库负责人签字：[signature]<br><br>2015年 01 月 02 日

保存期限：长期
