# Supplementary material for: PCSK9 promotes the progression and metastasis of colon cancer cells through regulation of EMT and PI3K/AKT signaling in tumor cells and phenotypic polarization of macrophages
Source: J Exp Clin Cancer Res. 2022 Oct 14;41:303. doi: 10.1186/s13046-022-02477-0 (PMC9563506; doi:10.1186/s13046-022-02477-0)
Supplement: Supplementary file 6 — Additional file 6. [file 13046_2022_2477_MOESM6_ESM.pdf]

济南市中心医院实验动物福利伦理审查批件

批件号	JNCH2021-79		
项目名称	基于代谢重编程重塑肿瘤微环境探讨 PCSK9 抑制剂调控结肠癌免疫应答的作用及机制		
项目来源	国家自然科学基金		
研究单位	济南市中心医院		
主要研究者	王潞		
审查类别	申报审查	审查方式	快速审查
批准文件	申报书		

审查意见

该项目组提交的课题中关于动物实验的方案，经我单位实验动物福利伦理委员会的审核，符合实验动物与动物实验福利、伦理的要求，同意申报。

济南市中心医院实验动物福利伦理审查委员会

2021 年 3 月 10 日

济南市中心医院 实验动物福利伦理审查委员会 3701027596858
